# Recent stabilization of agricultural non-CO_2_ greenhouse gas emissions in China

**DOI:** 10.1093/nsr/nwaf040

**Published:** 2025-02-13

**Authors:** Yuanyi Gao, Zimeng Li, Songbai Hong, Lijun Yu, Shihua Li, Jing Wei, Jinfeng Chang, Yao Zhang, Wen Zhang, Wenping Yuan, Xuhui Wang

**Affiliations:** Institute of Carbon Neutrality, Sino-French Institute for Earth System Science, College of Urban and Environmental Sciences, Peking University, Beijing 100871, China; Institute of Carbon Neutrality, Sino-French Institute for Earth System Science, College of Urban and Environmental Sciences, Peking University, Beijing 100871, China; School of Urban Planning and Design, Shenzhen Graduate School, Peking University, Shenzhen 518055, China; Key Laboratory of Earth Surface System and Human-Earth Relations, Ministry of Natural Resources of China, Shenzhen Graduate School, Peking University, Shenzhen 518055, China; State Key Laboratory of Atmospheric Boundary Layer Physics and Atmospheric Chemistry, Institute of Atmospheric Physics, Chinese Academy of Sciences, Beijing 100029, China; School of Atmospheric Sciences, Southern Marine Science and Engineering Guangdong Laboratory (Zhuhai), Sun Yat-sen University, Zhuhai 519082, China; School of Atmospheric Sciences, Southern Marine Science and Engineering Guangdong Laboratory (Zhuhai), Sun Yat-sen University, Zhuhai 519082, China; College of Environmental and Resource Sciences, Zhejiang University, Hangzhou 310058, China; Institute of Carbon Neutrality, Sino-French Institute for Earth System Science, College of Urban and Environmental Sciences, Peking University, Beijing 100871, China; State Key Laboratory of Atmospheric Boundary Layer Physics and Atmospheric Chemistry, Institute of Atmospheric Physics, Chinese Academy of Sciences, Beijing 100029, China; Institute of Carbon Neutrality, Sino-French Institute for Earth System Science, College of Urban and Environmental Sciences, Peking University, Beijing 100871, China; Institute of Carbon Neutrality, Sino-French Institute for Earth System Science, College of Urban and Environmental Sciences, Peking University, Beijing 100871, China

**Keywords:** greenhouse gas, agriculture, cropland, livestock, climate change

## Abstract

Agriculture emerges as a prominent contributor to CH_4_ and N_2_O emissions in China. However, estimates of these two non-CO_2_ greenhouse gases (GHGs) remain poorly constrained, hindering a precise understanding of their spatiotemporal dynamics and the development of effective mitigation strategies. Here, we established a consistent estimation framework that integrates emission-factor approach, data-driven models and process-based biogeochemical models, to identify the magnitudes, spatial variations, and long-term trends of agricultural non-CO_2_ GHG emissions in China's mainland from 1980 to 2023. Over the study period, the average total agricultural non-CO_2_ GHG emissions amounted to 722.5 ± 102.3 Tg CO_2_-eq yr^−1^, with livestock CH_4_, cropland CH_4_, cropland N_2_O and livestock N_2_O contributing 41% (297.4 ± 64.3 Tg CO_2_-eq yr^−1^), 31% (225.0 ± 69.6 Tg CO_2_-eq yr^−1^), 18% (130.6 ± 9.4 Tg CO_2_-eq yr^−1^) and 10% (69.4 ± 20.2 Tg CO_2_-eq yr^−1^), respectively. Approximately 70% of these emissions were concentrated in the eastern region beyond the Hu Line, with emission hotspots identified in South-central China, East China, and the Sichuan Basin. Our analysis revealed three distinct temporal stages of total emissions during the study period: rapid growth (1980–late 1990s), slow growth (late 1990s–middle 2010s), and a stabilization stage (since the middle 2010s). These stages reflect the evolving trajectory of agriculture in China, from the expansion of agricultural yields, to the transformation of agricultural practices, and ultimately the pursuit of sustainable development. However, the temporal trajectory of emissions varied significantly across different regions, highlighting divergent levels of agricultural development. This study presents a comprehensive, gridded, and consistent estimate of agricultural non-CO_2_ GHG emissions in China, offering valuable insights for policymakers to develop tailored strategies that adapt to local conditions, enabling effective emission reduction measures.

## Teaser

This study established a consistent estimation framework that integrates multiple methods to identify the spatiotemporal variations of agricultural CH_4_ and N_2_O emissions in China from 1980 to 2023. It revealed three distinct temporal stages of total emissions, reflecting the evolving trajectory of agriculture in China.

## INTRODUCTION

Agriculture, while nourishing 8.1 billion people, emerges as the largest single source of non-CO_2_ greenhouse gas (GHG) emissions, contributing to ∼31% of methane (CH_4_) and 52% of anthropogenic nitrous oxide (N_2_O) emissions [1,2]. Over the past decades, the rapid growth of global population and changes in dietary patterns have led to significant increases in agricultural production as well as GHG emissions, particularly in relation to CH_4_ and N_2_O [3,4]. Moreover, the demand for agricultural products is projected to increase throughout this century [5]. This trend will lead to further emission increases, posing a great challenge in achieving the target of limiting global temperature rise to 1.5°C [6]. To alleviate this burden, it is imperative to implement effective mitigation strategies to reduce non-CO_2_ GHG emissions from agriculture [7]. At present, 74% of countries have communicated their nationally determined contributions (NDCs) including GHG reduction in the agricultural sector [8], but the targets for non-CO_2_ GHG emissions from agriculture remain vague, with unclear details on reduction amounts, subsectors, and practices [9]. This gap is particularly notable in populous emerging economies like China, partly due to the limited availability of comprehensive estimates regarding non-CO_2_ GHG emissions from various agricultural activities.

China, as one of the most populous countries and largest agricultural producers globally [10], undoubtedly plays a significant role in global agricultural non-CO_2_ GHG emissions. For instance, a study based on inventory estimates and biogeochemical models suggested that China, with its rice paddy covering one-fifth of the global total, accounts for ∼22%–38% of CH_4_ emissions from rice paddies [11]. Similarly, China houses highly intensified and mix-farming livestock systems [12], serving 22% of global meat production [13]. They also contribute a considerable proportion of CH_4_ and N_2_O emissions, especially given the relatively lower forage quality and larger nutrient loss compared to the livestock systems in the United States and the European Union [14]. In addition, to feed 18% of the world's population with only 10% of cultivated area, China's mean fertilization rate stands at 191 kg N ha^−1^, which is three times larger than the global average [15]. The combination of a vast cultivated area and extensive N fertilizer use has made China the largest agricultural source of N_2_O emissions [1]. Apart from the immense magnitude, China's agricultural non-CO_2_ GHG emissions have been found to undergo a rapid increase over the past few decades [1,16], which largely neutralized the carbon sink in terrestrial ecosystems in China [17]. Therefore, a comprehensive and detailed estimation of non-CO_2_ GHG emissions from various agricultural activities in China, encompassing their magnitude, spatial distributions and temporal trends, will play a crucial role in supporting China's climate mitigation strategies and serve as a benchmark for other economies.

While existing assessments have provided valuable insights into the contribution of different production activities to agricultural non-CO_2_ GHG emissions in China, there still exist several limitations. These limitations stem from difficulties in encompassing the diverse range of crop and livestock species, the evolution of farming systems, and the influence of climate and soil conditions as well as management practices. For example, the intensification of agricultural systems in China, along with increased fertilizer availability and lower prices, has reshaped N fertilizer use patterns, thereby posing challenges in nitrogen-related emissions estimates. Additionally, the integrated impacts of various factors such as climate and soil conditions, and management practices were not adequately characterized in previous studies, leading to substantial uncertainty in agricultural GHG emission estimates. Indeed, various studies have shown significant variations in the magnitude of non-CO_2_ GHG emissions [18,19], and different temporal trends have even been observed in certain subsectors, particularly regarding livestock N_2_O emissions. There is a noticeable inconsistency in the literature regarding whether there has been a continuous increase in N_2_O emissions from the livestock sector over the past two decades [20]. These discrepancies are primarily attributed to different methodologies, input data, and emission factors (EFs) across studies. Furthermore, previous estimations of non-CO_2_ GHG emissions for different subsectors were usually based on divergent data sources (e.g. climate, soil and land use type), which could potentially undermine the comparability among datasets and induce uncertainties. Particularly, there are material flows shared among subsectors in agriculture. For example, the forage cultivated on cropland serves as feed for livestock, while manure produced by livestock is used as fertilizer which goes back to the cropland. However, these linkages are not well quantified due to separate estimations and divergent data sources in each subsector. Therefore, more efforts are needed to achieve consistent and reliable estimates of non-CO_2_ GHG emissions in China's agricultural sub-sectors.

Here, following the IPCC 2019 refinement guidelines [21], we established a highly consistent and reliable framework incorporating Tier 2 and Tier 3 methodologies for estimating agricultural non-CO_2_ GHG emissions (Fig. S1). In this framework, we made efforts to overcome the shortcomings identified in the aforementioned studies. Specifically, two process-based models, CH4MOD and IBIS-CH_4_, have been employed to quantify CH_4_ emissions from rice cultivation. We developed crop-specific machine learning models for ten crop types to estimate N_2_O emissions in cropland based on a comprehensive dataset of 1705 field observations. In addition, we established a multi-source dataset incorporating official and crowdsourced data to generate detailed spatial distributions of livestock CH_4_ and N_2_O emissions. The estimations of these four subsectors were based on consistent data sources, including climate data, cropland distributions, and agricultural production information from the *China Agriculture Yearbook*. We further integrated the manure produced by livestock and its corresponding application in cropland to achieve consistent estimates of non-CO_2_ GHG emissions from both cropland and livestock. Note that our estimates combined CH_4_ with N_2_O based on their global warming potential (GWP, in CO_2_ equivalent, 27 for CH_4_ and 273 for N_2_O) at a 100-year horizon [22] to assess the overall greenhouse effect. Note also that the adoption of GWP100 to calculate CO_2_ equivalent is not fundamentally scientific but depends on a policy perspective. Utilizing this comprehensive framework, we identified the magnitude, spatial distribution and temporal trends of agricultural non-CO_2_ GHG emissions in China over the period 1980–2023. This study provides valuable guidance for policy-making aimed at promoting sustainable agriculture in China, and its findings can also be applicable to other countries striving to ensure food security while reducing GHG emissions from the agricultural sector.

## RESULTS

### Comparisons with existing estimates

**Figure 1.**
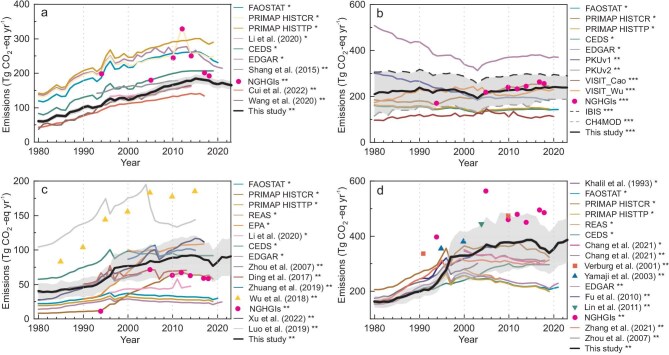
Comparisons of our results with existing estimates. (a) N_2_O emissions from cropland. (b) CH_4_ emissions from rice cultivation. (c) N_2_O emissions from livestock. (d) CH_4_ emissions from livestock. The symbols *, **, and *** denote the application of tier 1, 2, and 3 methods, respectively, in the estimations. Shadows represent the uncertainty of this study. Tier 1 methods rely on default emission factors, while Tier 2 and Tier 3 methods are based on more detailed, nationally derived information, with Tier 3 approaches possibly encompassing advanced models and resolved activity data. The uncertainty was quantified in each sector through uncertainty range provided by the IPCC, or the coefficient of variation across various models and activity data we used. More details about the methodology used in the datasets can be found in Tables S1–S4.

To validate our dataset, we first conducted a comprehensive comparison between our results with existing estimates. Our estimates for each subsector generally exhibited consistent temporal dynamics with previous studies, and the magnitudes fell within the middle range of them (Fig. 1). However, it is important to note that substantial discrepancies were observed among existing studies. Specifically, our estimate of N_2_O emissions from cropland aligned well with studies based on data-driven emission factors (e.g. Ref. [23,24]), but was much lower than the inventories using IPCC default EFs (e.g. Emissions Database for Global Atmospheric Research (EDGAR) [25], Potsdam Realtime Integrated Model for probabilistic Assessment of emissions Paths (PRIMAP) [26], Food and agriculture data (FAOSTAT) [27]) (Fig. 1a, Table S1). Our results also showed lower estimates than National Greenhouse Gas Inventories (NGHGIs) for the years 2005, 2010, 2012, 2014, 2017 and 2018 [28].

For the cropland CH_4_ emissions, two models used in our study presented consistent temporal dynamics but varied in magnitude, with IBIS-CH_4_ yielding higher results than CH4MOD (Fig. 1b). The synthesized results reported in our study (i.e. the average estimates from IBIS-CH_4_ and CH4MOD) showed good agreement with NGHGIs and other biogeochemical models such as VISIT [29]. When compared to the inventory-based estimates, our model-based result was comparable to estimates containing regional rice paddy information such as the PKU dataset [30] and NGHGIs. It is noteworthy that there were large differences among the global datasets FAOSTAT, EDGAR and Community Emissions Data System (CEDS), although their activity data were all obtained from the FAOSTAT [31].

Good agreements between our results and NGHGIs were also observed in terms of livestock CH_4_ emissions (Fig. 1d). Nevertheless, it is noteworthy that some disparities emerged in the trends in livestock CH_4_ emission since the mid-1990s among different studies. Some estimates (e.g. NGHGIs, CEDS, Fu *et al.* [32]) supported our findings, indicating that CH_4_ emissions from livestock experienced rapid growth until the beginning of this century, followed by a slower growth, while other studies (e.g. FAOSTAT, EDGAR, Zhang *et al.* [33]) have identified a decline in CH_4_ emissions since 2000. The difference mainly stemmed from CH_4_ emissions from enteric fermentation in ruminants (Fig. S2). Moreover, we identified that the observed emission differences around the years 1996–1997 from studies are primarily due to discrepancies in the sources of livestock statistical data (Fig. S3). This issue of data disaggregation within livestock statistics has also been noted by earlier research [34]. In our analysis, we adopted the data from the census year of 1996 as a baseline and harmonized information from multiple statistical yearbooks to ensure a more comprehensive and reliable evaluation.

The emissions of livestock N_2_O exhibited considerable discrepancies among different studies, with variations of up to more than 10-folds between various estimates (Fig. 1c). Nevertheless, our results in this subsector fell within the middle range among all the estimates. The estimates in this study were slightly lower than CEDS but slightly higher than NGHGIs (except for the year 1994), and all three datasets exhibited consistent temporal dynamics. In contrast, FAOSTAT and EDGAR showed much lower estimates and weak interannual variations. These comparisons serve as a reference for the credibility of our estimates, which fall within an acceptable range of uncertainty, aiding in a better understanding of the spatiotemporal variations and potential drivers of emissions.

### Spatial variations of agricultural non-CO_2_ GHG emissions in China

**Figure 2.**
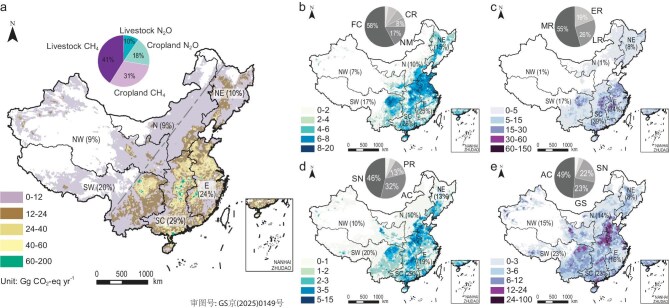
Spatial patterns of averaged agricultural non-CO_2_ GHG emissions during 1980–2023. (a) Total emissions of the four subsectors in China's agricultural sector. The dashed gray line indicates the Hu (Heihe-Tengchong) Line, while the inset pie chart indicates the proportion of each subsector. (b) N_2_O emissions from cropland. The pie chart shows the contribution of different nitrogen sources to cropland N_2_O emissions. FC, fertilization in cropland; NM, nitrogen mineralization; CR, crop residues. (c) CH_4_ emissions from rice cultivation. The pie chart shows the contribution of diverse sorts of rice paddies to CH_4_ emissions. MR, middle rice; LR, late rice; ER, early rice. (d) N_2_O emissions from livestock. The pie chart shows the contribution of a range of animal species to the N_2_O emissions. SN, swine; AC, all types of cattle; PR, poultry and rabbits. (e) CH_4_ emissions from livestock. The pie chart shows the contribution of various categories of key animals to the CH_4_ emissions. AC, all types of cattle; GS, goats and sheep; SN, swine. The division of China into six regions is as follows: NE, Northeast China; SC, South-central China; E, East China; N, North China; NW, Northwest China; SW, Southwest China. For other components not marked in the pie charts, see legends in Fig. 3d–g for details.

Averaged over the period 1980–2023, the total agricultural non-CO_2_ GHG emissions in China from the four subsectors amounted to 722.5 ± 102.5 Tg CO_2_-eq yr^−1^. Among the four subsectors, livestock CH_4_ emissions constituted the largest proportion with largest uncertainty, accounting for 41% of the total emissions (Fig. 2a). They were followed by CH_4_ emissions from rice paddy at 31%, the vast majority of which were contributed by middle rice (Fig. 2c). Cropland N_2_O emissions accounted for 18% of the total, which were primarily dominated by fertilizer-induced emissions (Fig. 2b). Livestock N_2_O emissions accounted for only 10% of the total emissions, with swine breeding contributing to nearly half of this total (46%, Fig. 2d).

The total non-CO_2_ GHG emissions exhibited considerable spatial heterogeneity, with significant difference between the two sides of the Hu Line (i.e. Heihe-Tengchong Line) (Fig. 2a). Large emissions were generally observed in South-central China and East China, which accounted for 29% and 24% of total emissions, respectively. The Sichuan Basin in Southwest China emerged as another hotspot for emissions, while scattered instances of high emissions were also observed in Northeast China. As a result, Southwest China and Northeast China contributed 20% and 10% of the total emissions, respectively. In contrast, North China and Northwest China, despite their large geographical area, accounted for only about 9% of the total emissions each.

Cropland N_2_O emissions showed similar spatial patterns to that of total non-CO_2_ GHG, with hotspots for emissions observed in South-central China, East China, Sichuan Basin and part of Northeast China (Fig. 2b). These areas coincided with high nitrogen fertilizer inputs, which directly contributed to elevated N_2_O emissions (Figs S4 and S5). These hotspots were also observed for N_2_O and CH_4_ emissions from livestock, although significant emissions were also observed in the western regions beyond the Hu Line (Fig. 2d and e), primarily contributed by ruminants rather than monogastric animals (Fig. S6). Higher CH_4_ emissions from rice paddy were generally observed in South-central China and East China (Figs 2c and S7). From the perspective of these two gases, the spatial distribution of CH_4_ shows a more distinct north-south pattern than N_2_O, primarily due to the dense distribution of rice paddies in southern China. Collectively, the observed patterns mentioned above provide compelling evidence of China's agricultural landscape, with emissions concentrated in the densely populated eastern regions, with the Hu Line serving as a distinct boundary.

### Long-term trends in agricultural non-CO_2_ GHG emissions over 1980–2023

**Figure 3.**
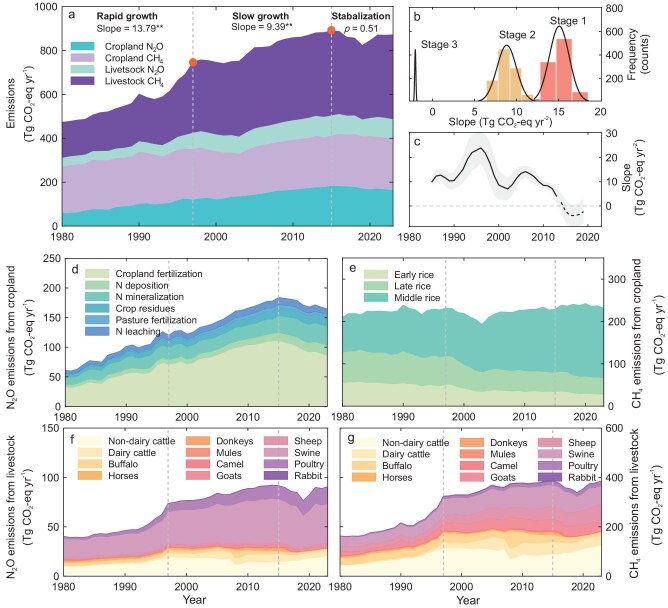
Temporal variations in agricultural non-CO_2_ GHG emissions of China during 1980–2023. (a) Temporal dynamics of total agricultural emissions. (b) Frequency distributions of the slopes for total emissions during the three stages. They were obtained from 10-year time series using the bootstrapping approach (1000-time random selections). (c) Consecutive changes in the slopes for the total emissions. The slopes were derived from 10-year moving windows, with central year from 1985 to 2019. Dashed line indicates the trend is not significant (*p* > 0.05). Temporal dynamics of (d) N_2_O emissions from cropland, (e) CH_4_ emissions from rice cultivation, (f) N_2_O emissions from livestock, and (g) CH_4_ emissions from livestock.

The total agricultural non-CO_2_ GHG emissions in China witnessed an increase from 474.5 ± 98.3 CO_2_-eq yr^−1^ in 1980 to 872.7 ± 99.5 Tg CO_2_-eq yr^−1^ in 2023, with an average annual growth rate of 10.77 Tg CO_2_-eq yr^−2^ (*p* < 0.05) (Fig. 3a). Notably, two distinct peaks were observed in 1997 and 2015, which divided the temporal changes in total emissions into three distinct stages: rapid growth (1980–late 1990s), slow growth (late 1990s–middle 2010s), and a stabilization stage (since the middle 2010s). Indeed, the first stage exhibited the largest positive trend, followed by the second stage, while the third stage showed a nonsignificant trend (Fig. 3b). This observation was further supported by the gradual decrease in the slopes of emissions derived from 10-year moving windows (Fig. 3c).

During the first stage (1980–1997), we observed a significant upward trend in total emissions (Fig. 3a and b), characterized by an average annual growth rate of 13.79 Tg CO_2_-eq yr^−2^ (*p* < 0.05). These substantial increases were primarily driven by CH_4_ and N_2_O emissions from livestock (Fig. 3f and g), as well as N_2_O emissions from cropland (Fig. 3d). In contrast, CH_4_ emissions from cropland exhibited minimal temporal trends and had a marginal impact on the three-stage transition, despite their significant contribution to the total emissions (Fig. 3e).

During the second stage (1997–2015), we observed a continued increase in total emissions, but the growth rate (9.39 Tg CO_2_-eq yr^−2^) notably decelerated compared to the first stage (Fig. 3a and b). This slowdown can be attributed mainly to the slower increases and even gradual stabilization of N_2_O and CH_4_ emissions from livestock (Fig. 3f and g). Meanwhile, cropland N_2_O emissions continued to increase at a comparable rate (3.70 Tg CO_2_-eq yr^−2^) to the first stage (3.80 Tg CO_2_-eq yr^−2^) (Fig. 3d). After 2015 (the third stage), the total agricultural non-CO_2_ GHG emissions exhibited very slight and nonsignificant trends (−1.89 Tg CO_2_-eq yr^−2^, *p* = 0.51) (Fig. 3a). That is, the total agricultural non-CO_2_ GHG emissions in China have ceased to increase since 2015. This stabilization was mainly attributed to the shift in trends of cropland N_2_O emissions since 2015, transitioning from rapid growth to slight decrease (−2.52 Tg CO_2_-eq yr^−2^) (Fig. 3a and d).

**Figure 4.**
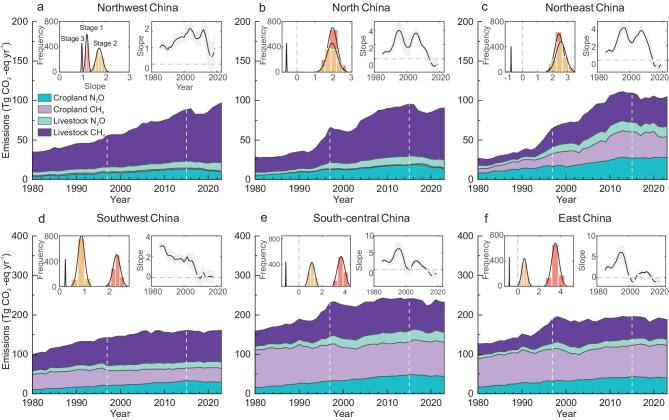
Regional specific trajectories of agricultural non-CO_2_ GHG emissions from 1980 to 2023. (a) Northwest China. (b) North China. (c) Northeast China. (d) Southwest China. (e) South-central China. (f) East China. The inset frequency distributions and line charts were obtained using a similar methodology as depicted in Fig. 3b and c.

Temporal changes in agricultural non-CO_2_ GHG emissions exhibited regional differences (Fig. 4). In the relatively more developed regions e.g. East China and South-central China, we found earlier stabilization processes since the late 1990s (Fig. 4e and f). Such stabilization was primarily driven by the slower growth of livestock CH_4_ and the decline of cropland CH_4_, both of which accounted for a substantial proportion of total emissions. In North China (Fig. 4b) and Southwest China (Fig. 4d), stabilization has started to occur since the 2010s, largely influenced by livestock CH_4_. In Northeast China, the stabilization of total emissions since the 2010s was mainly contributed by cropland rather than livestock (Fig. 4c). In Northwest China, however, a continued increasing trend was observed, with no obvious signs of stabilization (Fig. 4a), as also reported by previous studies [34,35]. This increase was predominantly driven by livestock CH_4_, highlighting the urgent need to implement efficient management practices in livestock farming to reduce the CH_4_ burden in Northwest China.

## DISCUSSION

### Improvements in estimation accuracy and consistency among sub-sectors

Through a comprehensive review of previous research, we have identified significant uncertainties and variations in existing estimates, with differences ranging from several folds to over 10-folds (Fig. 1). To address this, our study employed a hybrid Tier 2+ approach to use the highest tier from different sectors incorporating the latest advancements in understanding non-CO_2_ GHG emissions within each of the four subsectors. For the N_2_O emissions from cropland, we built crop-specific machine learning models based on a large dataset of 1705 field observations in this study, with ten different crop types considered. This approach addressed the limitations of previous research [36,37], which predominantly focused on staple crops and overlooked the consideration of cash crops. Furthermore, the utilization of machine learning allowed for a better understanding of the multifactor effects, surpassing the accuracy of employing regional average values [36,38] or multiple regression methods [37,39]. Consequently, the estimated N_2_O emissions from cropland in this study align well with those from other data-driven models, but are much lower than the datasets based on IPCC default EFs. It underscores the significance of considering spatial heterogeneity and variations among crop types, indicating that studies using uniform EFs fail to capture these factors accurately, leading to overestimations of N_2_O emissions from croplands in China.

Regarding CH_4_ emissions from rice cultivation, we used the Tier 3 approach including two process-based models (CH4MOD and IBIS-CH_4_) to capture the effects of varying climatic and edaphic conditions, with site-level emission records incorporated simultaneously. The two models we utilized displayed consistent dynamics and patterns but differed in magnitude (Fig. S7). The variation may be attributed to differences in model structure and parameters following the simplification and assumptions made regarding CH_4_ emission processes [40,41]. It is also important to note that the emissions from winter rice paddies were not considered in CH4MOD. Nevertheless, our estimates provide a much narrower range of uncertainty compared to the significant discrepancies observed among existing datasets, such as EDGAR and FAOSTAT, which indicate the improved constraint and reliability of our findings.

For the emissions of CH_4_ and N_2_O from livestock production, the IPCC (2019) Tier 2 approach was used in this study, with changes in livestock productivity and population dynamics being incorporated. Our estimates of N_2_O from livestock manure management are significantly higher than FAOSTAT and EDGAR. One potential reason for this discrepancy is that a Tier 1 approach was adopted in these datasets, which simplifies manure management conducted in different regions of China. Additionally, the disparities observed can be attributed to the updated representation of manure management systems in the IPCC (2019) guidelines [21]. Indeed, it is noted that globally, estimated emissions from manure management increased by ∼67% when transitioning from the IPCC (2006) guidelines [42] to the IPCC (2019) guidelines [43]. Regarding CH_4_ emissions from livestock enteric fermentation and manure management, our estimates are approximately twice as high as the results reported by FAOSTAT and EDGAR since the 2000s. They are also 25% higher than the estimates calculated using the version of IPCC (2006) approach and based on national livestock statistics. It is also important to note that our estimates closely align with NGHGIs, which utilized the most detailed national data, including county-level livestock populations and provincial livestock production characteristics, although the consistency in N_2_O estimates is not as strong as in CH_4_ estimates. This suggests that methodology and data sources in our study provide a more accurate representation of CH_4_ emissions from livestock activities.

In addition to the advancement made within each subsector, we took efforts to ensure the utilization of the same data sources for all four subsectors, enhancing consistency and comparability in our estimates. This is a significant improvement over previous studies, where such consistency was not achieved. Specifically, the same sets of agricultural data from China's *Agriculture Yearbook* including the area changes of cropland, plantation shifts, livestock herd population, livestock feed need, cropland and livestock production have been used in all subsectors. The same datasets for climate and soil conditions were also employed. Besides, we rebuilt the linkage between the animal manure and application of manure fertilizers. Specifically, the manure N used as fertilization input in cropland emission estimates was derived from the manure N output evaluated in livestock emission sectors based on the consistent data foundation. The link between N_2_O and CH_4_ emissions from cropland and livestock production identified in this study provides valuable insights for formulating more comprehensive and practical mitigation suggestions at high spatiotemporal resolutions.

### Social-economic factors underlying the spatiotemporal dynamics of agricultural non-CO_2_ GHG emissions

Our study identified three temporal stages of agricultural non-CO_2_ GHG emissions in China over the past decades: a rapid growth stage from 1980 to the late 1990s, a slow growth stage from the late 1990s to the middle 2010s, and a stabilization stage since the 2010s. The transitions of these three stages reflect the evolving trajectory of agriculture in China, from the expansion of agricultural yields, to the transformation and regulation of agricultural practices, and ultimately the pursuit of sustainable development. They are closely linked to demographic and economic dynamics [44,45], as well as government policy intervention [20,46,47]. During the first stage, China's population increased from 0.99 billion in 1980 to 1.24 billion in 1997 [48], creating a significant demand for crop and livestock products. To meet this increasing demand, the government implemented a series of policies to promote yield expansion, including raising grain purchase prices, reducing feed prices, and increasing investment in agricultural infrastructure [49,50]. The combination of population growth and policy incentives resulted in a surge in agricultural production and corresponding increase in GHG emissions. Additionally, as China's economy prospered, there was an increased demand for higher-quality, high-protein food, further intensifying the rise in livestock emissions. In fact, the sharpest increases during the first stage were observed in livestock CH_4_ emissions among the four subsectors (Fig. 3). This rapid growth phase was characterized by a near doubling of livestock production to meet increasing demand for animal products [44], which was also a direct response to the household contract responsibility system, under the umbrella of the ‘Vegetable Basket Project’ since the end of the 1980s, with the purpose of addressing urban food security [51].

Since 2000, population growth in China has been slowing down, and national agricultural policy has been gradually transformed, resulting in a slower growth of agricultural non-CO_2_ GHG emissions compared to the first stage. According to the National Bureau of Statistics of China, the population growth during the period 2000–2020 was approximately half as high as the growth observed from 1980–2020. Furthermore, the focus of agricultural support and protection has shifted towards enhancing agricultural efficiency and adhering to green ecological principles [52,53]. For instance, since the 2000s, there has been a notable transition towards the regional concentration of livestock farming, bolstered by national and industry standards for animal husbandry, such as the ‘Opinions of The State Council on Promoting the Sustainable and Healthy Development of Animal Husbandry’ launched in 2004 [44,54,55]. These standards were formulated to incentivize farmers to embrace standardized, large-scale practices. This consolidation has improved feed efficiency and facilitated the adoption of enhanced management techniques and technological advancements on a wider scale. Consequently, the slower increases in emissions from livestock played a significant role in the transition from the first to the second stage (Fig. 3). In contrast, the transition from the second to the third stage was mainly driven by decreasing cropland N_2_O emissions. This shift can be attributed to reduced nitrogen fertilizer inputs under ‘the Zero-Growth Action of Chemical Fertilizer Use’ policy implemented by the Ministry of Agriculture and Rural Affairs in 2015 [56]. Additionally, policies promoting the utilization of livestock waste since 2017 improved the appropriate use of manures, promoted nitrogen use efficiency, thereby reducing CH_4_ and N_2_O emissions from improper manure management [57]. This study demonstrated the effectiveness of these policies in China, which have contributed to the sustainable development of agriculture and have led to the stabilization, and even slight decrease, of agricultural non-CO_2_ GHG emissions.

Our results also highlighted the significant spatial heterogeneity in the trajectories of agricultural non-CO_2_ GHG emissions in China (Fig. 4). We observed earlier stabilization processes of emissions in more developed regions such as East China and South-central China, followed by South-west, North, and Northeast China. However, emissions in Northwest China continue to grow at present. This pattern may indicate an imbalance in regional agricultural development. Furthermore, we observed that the increase in cropland CH_4_ emissions in Northeast China was offset by the decreases in South-central and East China (Fig. 4), which are in line with the changes in harvested rice area. It suggests that spatial heterogeneity in the trajectories of emissions may also have roots in the adjustment in regional agricultural structures.

### Implications for policy-making to mitigate agricultural emissions

As a typical and major agricultural country, China's agricultural emissions account for ∼40% of the national total anthropogenic non-CO_2_ GHG emissions [58], with an 84% increase from 1980 to the present, as shown in this study, underscoring the urgent need to reduce emissions in the agricultural sector. In this study, the gridded maps of emissions from each subsector not only identify the hot spots of emission regions and agricultural activities, but also track the significant shifts in the spatial patterns of emissions. For instance, we clearly show the northward shift of livestock emission patterns due to pig breeding, aligning with the national strategy to stabilize and reduce the pig breeding scale in the southern regions concerning environmental pollution [59]. Moreover, the ruminant shifts towards the southwest and north plain correspond to the regions with favorable grassland resources and climatic conditions for ruminant farming [54]. Additionally, our maps highlight the increasing contribution from intensively fertilized vegetable and fruit cultivation areas. Our detailed emission patterns could provide substantive insights for making subsector-specific and region-specific measures to reduce agricultural non-CO_2_ GHG emissions.

Both cropland and livestock subsectors hold considerable potential for mitigation through increasing nutrient use efficiency, as the nutrient loss and GHG emissions in China's livestock are higher than those in both the United States and European Union [44], and intensity of fertilizer application in China's cropland far exceeds the global average [60]. When it comes to gases, CH_4_ accounts for 72% of the total emissions, implying the urgent priority of reducing CH_4_ emissions in the agriculture sector. The CH_4_ emissions from livestock, especially ruminants, contributing to ∼41% of the total emissions and half of the total increases over the past four decades (Fig. S8), offer the greatest mitigation potential via scientific feeding and manure management practices [13]. Previous estimates found that rice production has the largest GHG emission intensity among cereals [61]. As the world's largest rice production and consumption country [41], China's CH_4_ emissions from rice cultivation account for 31% of total agricultural non-CO_2_ GHG emissions. Therefore, improved management of paddy fields orienting climate change mitigation holds significant potential for the future [11].

Regarding the subsector of N_2_O emissions from cropland, it is predominantly driven by the input of nitrogen fertilizer. Indeed, the restriction of N fertilizer application policy has capped the abuse of fertilizers in nitrate-vulnerable areas, resulting in a significant decrease of national cropland N inputs (Fig. S9), which is in line with the decline in cropland N_2_O emissions since 2015 [24]. Enhancing nitrogen use efficiency in China's cropland through the development of innovative technologies and practices shows great potential for achieving emission reductions while ensuring food security [62]. Our estimates also indicate that the turning point of livestock CH_4_ and N_2_O emissions in the 2010s coincided with the implementation of the national ‘Livestock and Poultry Manure Utilization Action Plan (2017–2020)’ policy in 2017, which controlled manure discharge and improved the manure-recycle ratio in China [57]. Furthermore, studies have shown that increasing the proportion of manure in fertilizer application can reduce N_2_O emissions without reducing the overall nitrogen input [63]. Therefore, the integration of livestock and crop farming practices, such as the strategic relocation of livestock farms based on cropland distribution, would facilitate the efficient and effective recycling of manure nutrients, especially in the high-emission, mixed farming regions of eastern China. This approach would lead to reduced emissions in both cropland and livestock sectors, and would also minimize emissions associated with the transportation of manure and forage [64]. Overall, all the potential policies should adapt to local conditions, considering the high spatial heterogeneity in the composition and temporal dynamics of agricultural non-CO_2_ GHG emissions in China. Nevertheless, improving nutrient use efficiency and reducing food waste are both essential choices for promoting the reduction of agricultural emissions in all regions.

### Limitations and future directions

Despite the significant improvement of the new methodology, revised parameters, and sophisticated activity information used in this study, there are remaining uncertainties and limitations in our study that need to be noted. First, we focused on production-based emissions within the farm gate, while CH_4_ and N_2_O emissions from aquaculture, crop residue burning, leakage from biomass pools, and other similar sources were not considered. Second, while most of the activity data utilized in this study were obtained from the national to county-level statistical system, with low uncertainties, some data regarding the distribution of activities were not available on a yearly basis and required certain assumptions. For instance, when estimating the livestock CH_4_ and N_2_O emissions, we disaggregated provincial and county-level livestock numbers to the grid level, leveraging gridded maps provided by the Gridded Livestock of the World (GLW3 [65] and GLW4 [66]) for the years 2010 and 2015, respectively. The assumption was made that the spatial patterns of livestock between 1980 and 2023 shifted at a constant rate between these base years, which may not accurately reflect the actual situation. Additionally, the uncertainty in livestock emissions post-1996 can be primarily attributed to the increased diversity of available data and the changes in statistical methods employed in data gathering. This, along with a modest increase in EFs (Figs S10 and S11), has introduced additional variability into our results. Furthermore, it should be noted that the region-specific ratios of manure usage referred from livestock excretion and agro-climatology classification applied in this study may not accurately capture the spatial heterogeneity of manure practices, particularly in regions like western China, potentially leading to uncertainties. Third, while the use of process-based models has advanced our understanding of critical emission processes, the limited availability of observations for model validation introduces uncertainties in the outputs. Another limitation in our CH_4_ emissions modelling from rice paddies is the insufficient incorporation of some changing agricultural practices. For example, dry-direct-seeding and reduced flood irrigation have gained in popularity in central China in recent years [67–69]. Neglecting these changes may introduce uncertainty to CH_4_ emission estimates. Since the proportion of rice cultivated through direct-seeding remains relatively small [70], we did not include this method and its impact in our current simulation. However, future model improvements should prioritize the incorporation of the evolving practices in rice cultivars, planting, as well as irrigation to reduce the uncertainties in simulations [71]. Fourth, the data-driven estimates of cropland N_2_O emission factors rely heavily on the available observation data, incorporating the concept of space-for-time substitution and have limited understanding of the mechanisms underlying microbial N_2_O production. In addition, despite the optimal data sources of chemical fertilizer and manure that have been adopted in this study, which account for a large proportion of cropland nitrogen input and N_2_O emissions, we recognize that the inconsistency among different datasets used for other N sources (e.g. the deposited N obtained from the HaNi dataset [71], the mineralized N adopted from Ref. [72] based on the N mineralization rate derived from the Community Land Model 5.0 [73]) may also introduce some uncertainties into our estimates.

Future efforts should focus on depicting a more comprehensive picture of GHG emissions from agricultural systems, and improving understanding of the mechanisms behind N_2_O and CH_4_ emissions from soil and livestock microorganisms, which would contribute to the development of more accurate and reliable process-based models. Expanding the accounting boundaries to include emissions from additional processes, both within and beyond the farm gate, would offer a more systematic perspective on the characteristics of agricultural emissions. Additionally, it is necessary to increase the availability of observation data, particularly in regions and for parameters that currently lack sufficient data. This will lead to improved accuracy and reliability in estimating emissions, and ultimately enhance our overall understanding of agricultural GHG emissions in China.

## METHODS

This study estimated agricultural non-CO_2_ GHG emissions for four subsectors: cropland N_2_O, cropland (rice paddy) CH_4_, livestock N_2_O (manure managements) and livestock CH_4_ (enteric fermentation of ruminants and manure managements) following the IPCC Tier 2∼3 (2019) approaches modified with additional sectoral information (Fig. S1). Estimations of the four subsectors were based on a consistent data foundation e.g. *China Agriculture Yearbook* from 1980 to 2021 [70], and data from the National Bureau of Statistics of China [48], as well as the same climate maps, crop maps and land use data. Emissions series for each subsector were established annually from 1980 to 2023 at a spatial resolution of 0.1° ∗ 0.1° in China's mainland (Taiwan province was not included). Given the limited availability of data for the latest year, the emissions for 2023 in China were estimated based on gridded activity data extrapolated from the previous 5 years and static EFs of the year 2022. Methods for each subsector are summarized as below and detailed methods are described in Supplementary Section S1.

### N_2_O emissions from cropland

N_2_O emissions from cropland were estimated by multiplying EFs with different nitrogen loads, including N fertilization, N deposition, N mineralization, N in crop residues, N fertilization in pasture and N leaching. Crop-specific random forest models were employed, based on 1705 field observations, to derive gridded EFs for various factors such as N fertilization, N deposition, N mineralization, N in crop residues and N fertilization in pasture. For each RF modeling, we randomly divided 70% of the measurement records to train the models and the remainder served as testing data to avoid overfitting. The finally constructed RF models exhibited satisfying performance in simulating EFs for various crops, with total R^2^ being 0.87 and RMSE being 0.32, which were considered as qualified for the EF prediction. It should be noted that both synthetic N and manure N have been included for N fertilization in cropland, and the manure N used as fertilization input was derived from the manure N output evaluated in livestock (See Supplementary Section S1.3 and S1.4). For manure fertilizer produced after manure management, its application to cropland is only considered in regions classified as cropland-based livestock production systems, based on the agro-climatology classification by Herrero *et al.* [74], which distinguishes solely livestock systems and mixed crop-livestock farming systems according to livestock feed sources, temperature and the length of the growing period. In addition, the N_2_O emissions resulting from N leaching were calculated by adopting leaching coefficients from Ref. [75].

### CH_4_ emissions from rice paddy

CH_4_ emissions from rice paddies were simulated using two biogeochemical models, CH4MOD [76] and IBIS-CH_4_ [77]. These models aim to accurately simulate CH_4_ production and oxidation by integrating key microbial mechanisms, such as anaerobic fermentation and homoacetogenesis, hydrogenotrophic methanogenesis, acetoclastic methanogenesis and methanotrophy. Both models are driven by the same climate and crop phenology data with consistent crop distribution in this study, and three CH_4_ transport processes from soil to atmosphere (i.e. diffusion, plant-mediated transport and ebullition) have been included. However, there are also differences in their methodological details and priorities. The CH4MOD is a semi-empirical model which consists of two modules, the derivation of the methanogenic substrates and the processes of methane production and emission. It has been validated across 94 sites covering different regions and growing seasons in China, ensuring robust performance for regional simulations. It aims at simulating the growing rice biomass which is used as the key variable in calculating the root exudates and the fraction of the methane emissions from plants and bubbles, daily changes in the soil redox potential (Eh) were also calculated with differential functions, according to various water manipulations in the rice paddies (see S1.2.1). The IBIS-CH_4_ model is a process-based land ecosystem model, which has been validated at 16 rice field sites and 50 wetland sites worldwide. To estimate CH_4_ emissions from rice paddies, it has modified the structures of topsoil layers and the corresponding root fractions, to align with the typical root distribution pattern in rice paddies (see S1.2.2). These adaptations confer distinct advantages to each model. Therefore, both models were used in this study to estimate the CH_4_ emissions from rice cultivation in China, enhancing the credibility and accuracy of our results compared to data-driven methods.

### CH_4_ emissions from livestock

CH_4_ emissions from livestock include two sources: enteric fermentation and manure management. We considered 12 major livestock categories including dairy cattle, non-dairy cattle, buffalo, sheep, goats, swine, camel, mules, donkeys, horses, poultry and rabbit. The estimates were primarily based on the IPCC 2019 (Vol. 4, Chapter 10) Tier 2 method. Enteric fermentation CH_4_ emissions from main ruminants such as dairy cattle, non-dairy cattle, buffalo, sheep and goats were estimated based on the gross energy intake of livestock (GE) and a conversion factor, *Y_m_*, calculated from the digestibility of feed (DE) (see S1.3.2).

CH_4_ emissions from manure management were estimated based on the volatile solids (VS) from livestock excretion for different manure management systems (Table S5). The estimation was made at the grid cell level with the gridded VS following the annual livestock distributions in China. In describing manure management in China, we updated the categorization proposed from peer-reviewed literature [20,78] and the NGHGIs (see S1.3.3).

For the activity data, besides the provincial data collected from the consistent source of regular statistical data, the national census data (for the years 1996, 2006 and 2016) [79] has been used to modify the regular statistical data using an average velocity trend method, the county-level livestock information from three years (1992, 2012 and 2017) [80–82] and gridded livestock distribution (5arcmin) from GLW have been incorporated with our provincial data aimed to depict a more detailed spatial pattern of livestock emissions in China (see S1.3.1).

### N_2_O emissions from livestock

The dominant source of N_2_O emissions from livestock production is manure. The emissions were estimated using manure N excretion and EFs at different manure management systems to account for N losses involving direct N-N_2_O emissions, indirect emissions from volatilized N-NH_3_ and leached N-NO_x_ (Tables S5 and S6). The manure N excretion was estimated from the N intake, based on gross energy intake (GE), and then subtracted the fraction utilized in livestock retention (see S1.4). Two parts of the indirect N_2_O emissions were estimated based on our estimation of N excretion and the default values of nitrogen loss coefficients from the IPCC (2019). It should be noted that N_2_O emissions from the manure field-application stage and fertilizer N applied to pasture were not contained in the livestock subsector but considered in cropland emissions (Table S7).

### Statistical analysis

Two prominent peaks in the series of agricultural non-CO_2_ GHG emissions were identified, occurring in the years 1997 and 2015 (Fig. 3a), which divided the series into three stages. The emission trends during each stage were identified as the slopes in linear regression. A bootstrapping sampling approach was employed to assess the robustness of our results. Specifically, the data of 10 years were randomly selected for regressions in each stage. Note that each stage was supplemented by an additional 3 years from the adjacent periods (i.e. 1980–2000, 1994–2018 and 2012–2023) during the bootstrapping to minimize the influence of the choice of the turning points. The random selections and corresponding regressions were repeated for 1000 times to generate frequency distributions of the slope in each stage (Fig. 3b). In addition, we further calculated the consecutive changes in the slopes of total emissions using a 10-year moving window, with the central year ranging from 1985 to 2019 (Fig. 3c). Analyses in Fig. 4 were conducted in similar ways.

### Uncertainty analysis

To assess the uncertainties in CH_4_ and N_2_O emissions from various sectors, we employed a Monte Carlo approach with 10 000 simulations, accounting for variations in activity data and emission factors (EFs). Given the scarcity of evaluation data for EFs, we utilized the standard deviation of regional EFs as provided by the IPCC for each sector. The uncertainty in activity data was defined by the coefficient of variation across all datasets considered.

For the estimation of N_2_O emission from cropland, the standard deviation among emission estimates from five fertilizer input datasets (Table S8) has been accounted. In the livestock CH_4_ and N_2_O emissions, uncertainty primarily stems from discrepancies between the high spatiotemporal resolution dataset revised for this study and the national datasets referenced, including county-level datasets from peer-reviewed literature, provincial datasets from the *China Agriculture Yearbook* and National Statistical Bureau, and national results from international reports such as FAOSTAT. We assume uniform distributions for activity data and normal distributions for the EFs.

For CH_4_ emission estimates from rice paddies, the relative uncertainty was directly derived from the model structural uncertainty within two process-based models utilized in this study, calculated by dividing the standard deviation by the multi-model mean.

### Comparison with existing studies

We conducted a comprehensive literature review to examine existing studies on non-CO_2_ GHG emissions from China's agriculture. This allowed us to make a thorough comparison with our own estimates for each of the four subsectors. Well-known datasets such as NGHGIs, EDGAR, FAOSTAT and CEDS were included in our analysis. Detailed comparisons and information of datasets we used can be found in Tables S1–S4.

## Supplementary Material

nwaf040_Supplemental_File

## Data Availability

All data needed to evaluate the conclusions in this paper are presented in the paper and/or the Supplementary data. The agricultural emission dataset constructed by this study will be soon available publicly under the CNGHG2023 project (https://carbon.pku.edu.cn/). Additional ancillary data are available from the corresponding author upon reasonable request.
