# Supplementary material for: Recent stabilization of agricultural non-CO2 greenhouse gas emissions in China
Source: Natl Sci Rev. 2025 Feb 13;12(4):nwaf040. doi: 10.1093/nsr/nwaf040 (PMC12089786; doi:10.1093/nsr/nwaf040)
Supplement: nwaf040_Supplemental_File [file nwaf040_supplemental_file.docx]

**Recent stabilization of agricultural non-CO_2_ greenhouse gas emissions in China**

**Supplementary Information**

**Section S1: Supplementary methods**

The methodology used in this study are based on IPCC's guidelines for estimating GHG emissions, which categorized the methods into Tier 1, 2, and 3 approaches. Tier 1 uses default EF from previous studies, while Tier 2 and Tier 3 methods are based on more detailed, nationally derived information. Tier 3 approaches may also include more advanced models and activity data that are temporally and spatially resolved. Tier 2 and 3 methods were employed in this study.

**S1.1 N_2_O emissions from cropland**

For this subsector, N_2_O emissions from cropland were estimated using EF methods, considering six items: N fertilization, N deposition, N mineralization, N in crop residues, N fertilization in pasture, and N leaching (eq.1).

$E_{N_{2}O\_crop}=\sum_{i=1}^{6} EF_{i}\bullet N_{input_{i}}\bullet\frac{44}{28}$ (1)

where *E_N2O_crop_* represents the total N_2_O emissions from cropland soils (kg N_2_O per hectare); *i* ranges from 1 to 6, and represents N_2_O emissions from above items respectively; 44/28 is the conversion coefficient from N_2_O-N emissions to N_2_O emissions.

Regarding nitrogen inputs from cropland fertilization, we employed five datasets (Table S8), and the average of them was used in this study. Note that both chemical fertilizer and manure used in cropland were included in each dataset, following eq.2:

$N_{input1}=(N_{\text{rate}_{\text{chem}}}+N_{rate_{man}})\bullet\text{area}$ (2)

where *N_input1_* represents the nitrogen inputs in the form of fertilization (kg N_2_O-N); *N_ratechem_* and *N_rateman_* respectively represent the application rates of chemical N and manure N (kg N_2_O-N per hectare).

The data for nitrogen fertilization and sowing area during 1980-2023 were derived from existing datasets and modified with NBSC. In addition, nitrogen inputs in the form of deposited N and pasture fertilization (including chemical fertilizer and manure N input in pasture) were obtained from the HaNi dataset [1]. The crop residues N, leaching N and the mineralized N were from Liang et al. [2].

Gridded EFs for the first five items were simulated via machine learning models [3], while leaching coefficient was obtained from Zhou and Butterbach‐Bahl, 2014 [4]. Specifically, we compiled a comprehensive and detailed N_2_O-EF dataset including 1705 in-situ N_2_O emission observations and 1163 N_2_O-EF records from 221 peer-reviewed papers. Such an extensive dataset enabled a more detailed crop classification, with both staple grain and cash crops considered. Based on this dataset, we built crop-specific random forest models for 10 crop types, i.e., rice, wheat, maize, cotton, fruit, legume, oil plant, tea, vegetables and others. We established 30 models for each crop type, and their average values were reported as final output EFs. The constraints of field records have improved our simulated EFs and the RF-based predictions exhibited better consistency with the synthesized records than previous studies [5, 6].

**S1.2 CH_4_ emissions from rice cultivation**

Two process-based models, namely CH4MOD and Integrated Biosphere Simulator CH_4_ (IBIS-CH_4_) model, were utilized in this study to simulate CH_4_ emissions from rice paddy. The two models were driven by consistent meteorological, phenological and yield data. Note that the rice paddy distribution used for CH_4_ emission estimation was the same as that used for N_2_O emission estimation in S1.1. The structure and parameters of the models are described below:

**S1.2.1 CH4MOD**

The CH4MOD is a semi-empirical model that simulates the daily methane emissions from rice paddies under various agricultural practices. This model consists of two modules: the derivation of the methanogenic substrates and the processes of methane production and emission [7]. The former module simulates the production of the methanogenic substrates that are primarily derived from rice root exudation and added organic matter (i.e., crop residues and manure). The latter module simulates the methane production from the available methanogenic substrates and the fraction of emissions via rice plants and bubbles. The model used a logistic function with rice grain yield as the input to simulate the growing rice biomass that is a key variable in calculating the root exudates and the fraction of the methane emissions from plants and bubbles [8]. The daily changes in the soil redox potential were calculated with differential functions, according to various water manipulations in the rice paddies [9, 10]. The influences of other environmental factors, such as soil temperature and texture, on organic matter decomposition and methane production were expressed as specific coefficient functions [8].

Daily mean ambient air temperature is the only meteorological data required to drive the CH4MOD model. The daily mean air temperatures of each grid were derived from ERA5 with a resolution of 0.1°×0.1° [11].

The distribution and phenology maps of rice in China between 2017 and 2020 were generated by using a phenology-based method (the time-weighted dynamic time warping method) that combines optical and synthetic aperture radar images, and they had been constrained by provincial-level paddy field area data of China and validated by abundant field surveyed samples. The distribution of paddy fields before 2017 or after 2020 were consistent with the distribution maps of 2017 or 2020. The paddy field area of each pixel was calculated by the proportion of paddy field area of a target year to the paddy fields area of 2017 or 2020. The resolution of grid data is 0.1°×0.1°.

The crop yields were collected from the National Bureau of Statistics of China (NBSC) [12]. The straw return rates and manure after 2018 were provided by the Rural Energy and Environment Agency, Ministry of Agriculture and Rural Affairs. For data before 2018, the estimates were based on the China Rural Energy Yearbook [13, 14].

**S1.2.2 IBIS-CH_4_**

The IBIS-CH_4_ model aims to accurately simulate CH_4_ production and oxidation by integrating key microbial mechanisms, such as anaerobic fermentation and homoacetogenesis, hydrogenotrophic methanogenesis, acetoclastic methanogenesis, and methanotrophy [15]. The IBIS-CH_4_ model also includes production of CO_2_, O_2_, and H_2_ besides CH_4_, and three CH_4_ transport processes from soil to atmosphere: diffusion, plant-mediated transport, and ebullition. A critical component of the model is the water table depth (or the soil water content), which plays a vital role in demarcating the soil column into aerobic and anaerobic zones. These zones are crucial for the production and oxidation of CH_4_. In the initial configuration of the IBIS-CH_4_ model, methane related biogeochemical processes transpired across the top nine soil layers, encompassing a depth of 2 m. The structures of these layers included five layers of 0.1 m each, followed by four layers with depths of 0.2, 0.3, 0.5, and 0.5 m, respectively. The distribution of available carbon for methanogens across all soil layers were calculated using the dynamic root fractions determined by the IBIS model [16]. However, in our adaptation for rice cultivation, we acknowledge the predominant distribution of rice roots within the top 20 cm of the soil layers [17]. Consequently, we modified the root fractions in the top four soil layers to be 0.7, 0.2. 0.07, and 0.03, respectively, to align with the typical root distribution pattern in rice paddies.

IBIS-CH_4_ model was run based on consistent meteorological, phenological and distribution map with that used in CH4MOD.

**S1.3 CH_4_ emissions from livestock**

Livestock CH_4_ in this study only include emissions from enteric fermentation and manure management, which were estimated primarily based on IPCC 2019 (Vol. 4, Chapter 10) [18] Tier 2 method by multiplying the livestock activity data and region-specific EFs for different livestock categories. Specifically:

$E_{\text{CH}_{4}\text{\_ef}}=\sum_{i,P,\text{K}} (EFef_{i,P,K}\times(\sum_{L} N_{i,P,K,L}))$ (3)

$E_{\text{CH}_{4}\text{\_mm}}=\sum_{i,P,\text{K}} (EFmm_{i,P,K}\times(\sum_{L} N_{i,P,K,L}))$ (4)

where *E_CH4_ef_* (kg CH_4_ per year) is CH_4_ emissions from enteric fermentation; *P* represents the production system; *K* represents region; *L* represents live span structure of the animal type; *EFef_i,P,K_* is emission factor of enteric fermentation for the animal type *i* in the production system *P* of the region *K*; *EFef_i,P,K_* (kg CH_4_ per herd per year) is emission factor of enteric fermentation for the animal type *i* in the production system *P* of the region *K*; *N_i,P,K,L_* (herd) is the population number of animal type *i* with the age structure *L* in the production system *P* of the region *K*; *E_CH4_mm_* is CH_4_ emissions from manure management; *EFmm_i,P,K_* is the emission factor for the animal type *i* in production system *P* in region *K*.

**S1.3.1 Calculation and data sources on livestock activity**

The annual population for 12 livestock categories (including dairy cattle, non-dairy cattle, buffalo, sheep, goats, swine, camel, mules, donkeys, horses, poultry and rabbit) in each province of China during the period of 1980–2021 were derived from the China Agriculture Yearbook [19], and the population of main animal categories for 2022 were taken from NBSC [12]. Since the NBSC only provides the total number of cattle, the proportion of dairy cattle, non-dairy cattle and buffalo was extracted based on our results of the year 2021. Given the limited information of the latest year, the activity data for 2023 in China were extrapolated from the previous 5-year trend. It should be noted that the year-end live population and slaughtered population were both included in our estimation. Considering the large inconsistency between census year and regular statistical year, as well as between national and regional inventories [20], we revised the provincial annual activity data from statistical yearbook based on the three agricultural censuses in 1996, 2006, and 2016 [21], assuming that agricultural censuses have higher confidence than regular statistical yearbook. The average velocity trend method has been used to adjust regular statistical data [22] between the two censuses as follows:

$N_{yr,i,K}^{'}=N_{yr-1,i,K}^{'}\times\text{e}^{\text{ln}\frac{N_{yr,i,K}}{N_{yr-1,i,K}}+\frac{1}{10}\ln\frac{C_{yr_{n},i,K}}{N_{yr_{n},i,K}}}$ (5)

where *N^’^_yr,i,K_* is the revised annual livestock population in animal *i*, region *K*, year *yr*; *N_yr,i,K_* is the general statistical data of annual livestock population in animal *i*, region *K*, year *yr*; *C_yrn,i,K_* is the census data of annual livestock population in animal *i*, region *K*, census year *yrn*. Note that the first agricultural census in China was conducted in 1996, so we did not revise the data before 1996. Furthermore, we adopted the classification standard of traditional, medium and industrial production systems (*P*) derived from previous studies [23, 24] to further subdivide the livestock numbers.

We then downscaled the revised provincial activity data during 1980 to 2023 to grid scale with reference to the livestock distribution data at a finer spatial resolution. Firstly, available county-level livestock numbers for several years have been collected. Data for the year 1992 and 2012 were obtained from ref [25, 26], and 2017 from ref [27, 28], with more than 3,300 counties in mainland China have been surveyed under the second China Pollution Sources Census Committee. We interpolated county-level data for the remaining years of 1980-2023 based on the three-year data above, and further rescale the county numbers in each province to be aligned with the revised provincial activity data. Finally, we disaggregated county-level livestock numbers to the grid level, leveraging gridded maps provided by the Gridded Livestock of the World [29, 30] for the year 2010 and 2015, respectively. Specifically, for each county, we used the gridded distribution of GLW3 for downscaling the county-level data between 1980-2010, and that of GLW4 for 2015-2023. The assumption was made that the spatial patterns of livestock between 1980 and 2023 shifted at a constant rate between these three base years.

**S1.3.2 Calculation of enteric fermentation CH_4_ emission factor**

The EFs of enteric fermentation CH_4_ emissions from main ruminants such as dairy cattle, non-dairy cattle, buffalo, sheep, and goats were estimated adapted from IPCC Tier 2 algorithms as follow.

 (6)

where, *EFef* is primarily based on the gross energy intake of livestock (*GE*) and a conversion factor, *Y_m_*, calculated from the regional digestibility of feed (*DE*). The data for *Y_mi_* were determined by the feed quality, we evaluated it for each animal category referred from previous estimate [20]. The *GE* required for each livestock category can be estimated using the equation:

$GE=\left[\frac{\left(\frac{NE_{m}+NE_{a}+NE_{l}+NE_{work}+NE_{p}}{REM}\right)+\left(\frac{NE_{g}+NE_{wool}}{REG}\right)}{DE}\right]$ (7)

where, *GE* is the gross energy; *NE_m_* is the net energy required by the animal for maintenance; *NE_a_* is the net energy for animal activity; *NE_l_* is the net energy for lactation; *NE_work_* is the net energy for work; *NE_p_* is the net energy required for pregnancy; *REM* is the ratio of net energy available in a diet for maintenance to digestible energy consumed; *NE_g_* is the net energy needed for growth; *NE_wool_* is the net energy required to produce a year of wool; *REG* is the ratio of net energy available for growth in a diet to digestible energy consumed; *DE* is the digestible energy expressed as a percentage of gross energy.

The above variables can be estimated following equations 10.3–10.15 from the IPCC (2006) guideline, Main parameters that influence the *GE* include the live body weight (kg per head), weight gain (kg per head per day), milk production (kg per head), milk fat content (%), wool production (kg per head), lactation rate (%), gestation rate (%), feed digestibility (%) and work hour (h per day). We collected provincial body weight information and milk production data from peer review estimates and coerced by the ten years (2004−2013) survey data [20, 31-33] (Zhang et al., 2021, Fig. S3; Yu et al., 2018, Table S9; Xu et al., 2019, Table S3–5; Opio et al., 2013, Table B2–6). For the estimation of digestible energy, average values of 56.7%, 60%, 52.5%, 59.2% for dairy cattle, nondairy cattle, buffalo, and sheep (or goat) referred from National Greenhouse Gas Inventories (NGHGIs) [34] were used in this study. For other parameters, the recommended regional default numbers were used referred from the IPCC (2019) guideline.

**S1.3.3 Calculation of manure management CH_4_ emission factor**

The EFs of CH_4_ emissions from manure management, for all livestock categories, were estimated based on the volatile solids (*VS*) excreted by livestock, maximum methane production capacity for manure produced by livestock (*B_0_*), methane conversion factors (*MCF*) for each manure management system and each climate region, and the fraction of livestock manure handled using each animal waste management system (*AWMS*) in each region. Mathematically,

$EFmm_{i,P,K}=(VS_{i,P,K}\bullet365)\left[B_{0 (i,P,K)}\bullet0.67\bullet\sum_{S, K} (\frac{MCF_{K}}{100}\bullet AWMS_{i,P, S, K})\right]$ (8)

$VS=\left[GE\bullet\left(1-\frac{DE}{100}\right)+UE\bullet GE\right]\bullet\left(\frac{1-ASH}{18.45}\right)$ (9)

where, *VS_i,P,K_* is the volatile solid excreted in the animal type *i* in the production system *P* of the region *K*, which is derived based on our previous evaluation of *GE*, *DE* following eq.7, in which the *UE* is the urinary energy expressed as a fraction of *GE*, and *ASH* is the ash content of manure calculated as a fraction of the dry matter feed intake.

*MCF_K_* is the methane conversion factor in region *K*. We evaluated it based on the climate zones divided by the long-term average temperature in region *K*, we obtained mean annual temperature at the province level from the ERA5 [11].

*AWMS_i,P,S,K_* is the applying ratio of manure management systems from animal type *i* in the production system *P* under the manure management *S* of the region *K* (Table S5). The province level information about the *AWMS* for each livestock category in China was extracted from previous literature basing on field surveys and the NGHGIs [24, 34].

The *B_0_* of each livestock category *i* in the production system *P* of the region *K* was depended on information from NGHGIs (2014, Table 4-49).

**S1.4 N_2_O emissions from livestock**

**S1.4.1 Calculation of manure management N_2_O emission factor**

The N_2_O emissions from livestock manure managements were estimated based on IPCC Tier 2 approach, which mainly relate to animal nitrogen excretion, EF for N_2_O emissions, as well as volatilization and leaching factors. Activity data source was generally consistent to that used in the estimation of livestock CH_4_ emissions (Text S1.3.1). The direct N_2_O emissions were estimated using the following formula:

$E_{N_{2}O\_mm}=\left[\sum_{S} \left[\sum_{i, P,K} \left(\left(\sum_{L} (N_{i, P,K,L})\bullet Nex_{i, P,K}\right)\bullet AWMS_{i, S, P,K}\right)\right]\bullet EF_{N_{2}O(S)}\right]\bullet\frac{44}{28}$ (10)

where, *E_N2O_mm_* (kg N_2_O per year) is the direct N_2_O emissions from manure managements; *S* represents the manure management systems; *i* represents the animal type; *P* represents the production system; *K* represents region; *N_i,P,K,L_* (herd) is the population number of animal type *i* with the age structure L in the production system *P* of the region *K*; *N_ex(i,P,K)_* (kg N per herd per year) is the nitrogen excretion rate of animal type *i* in the production system *P* of the region *K*; *AWMS_i,S,P,K_* (%) is the fraction of total annual nitrogen excretion, of animal type *i* in a given manure management system *S* and the production system *P* of the region *K*. *EF_3(S)_* (kg N_2_O-N per nitrogen excretion per year) is the emission factor of direct N_2_O emissions from the manure management system *S*. Values of *EF_3(S)_* were synthesized from the IPCC (2006, the Table 10.21) and peer review estimate (Table S6) [24]. 44/28 is the conversion factor of N_2_O-N to N_2_O emissions.

The total amount of N excretion (*N_ex_*) from main livestock categories in each type of manure management system were estimated based on the gross energy intake (GE) (eq. 7), then subtracted the fraction utilized in livestock retention (eq. 12).

$N_{intake(i,P,K)}=\frac{GE_{i,P,K}}{18.45}\bullet\left(\frac{\frac{CP_{i,P}\%}{100}}{6.25}\right)$ (11)

 (12)

where, *GE_i,P,K_* is the gross energy intake of animal type *i* in production system *P* of region *K*, which is in line with the estimation in S1.3.2; *CP_i,P_* is the percentage of crude protein in the diet of animal type *i* in production system *P*, which were taken from defaults results of the IPCC (2019, Tables 10A.1–10A.4); *N_retention_frac(i,P,K)_* is the fraction of nitrogen intake that is retained by animal type *i* in production system *P* of region *K,* which is also derived from IPCC (2019, Table 10.20). For animals lack of *CP* estimation or the GE estimations, we estimated the *N_ex_* based on the average body weight (*TAM*) and the default nitrogen excretion rate *N_rate_* of animal type *i* in production system *P* of region *K* as following:

 (13)

The indirect emissions from volatilized N-NH_3_ and leached N-NO_x_ were estimated based on our N excretion and the default values of nitrogen losses coefficients from the IPCC (2019) Table 10.22 and the default values of EFs from the IPCC (2019) Table 11.3.

The coefficients of manure nitrogen applied to croplands, in different livestock systems, and livestock types are listed in Table S7 referred from peer review literature and Guidelines for integrated emission inventories of air pollutants and greenhouse gases, which is estimated in line with other utilization of manure nitrogen such as the nitrogen loss, the total manure managed for different livestock production systems, and managed manure to other use.

**Section S2: Supplement figures**


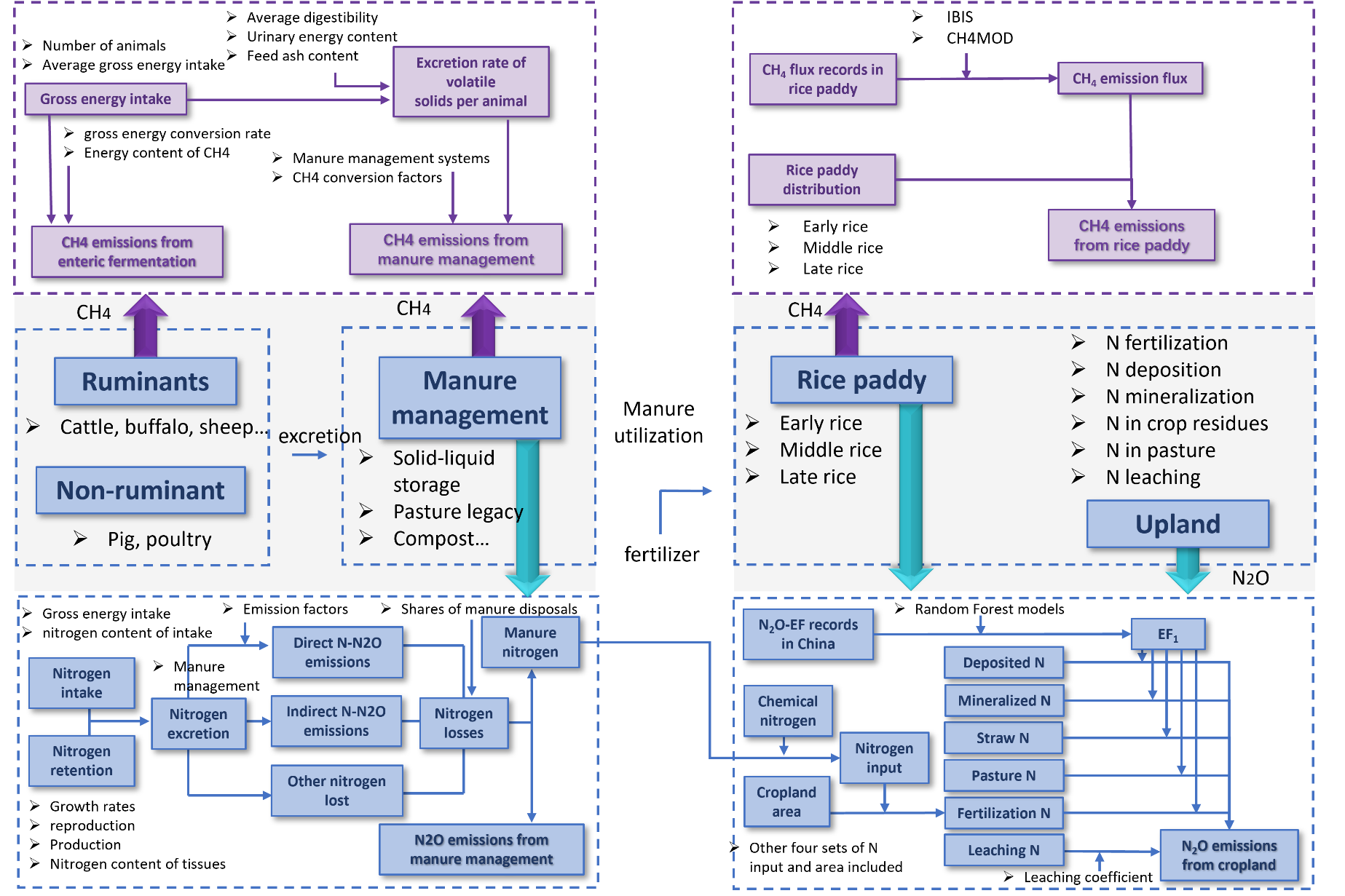


**Figure S1.** Methodological framework for estimating emissions in each subsector.

a

b


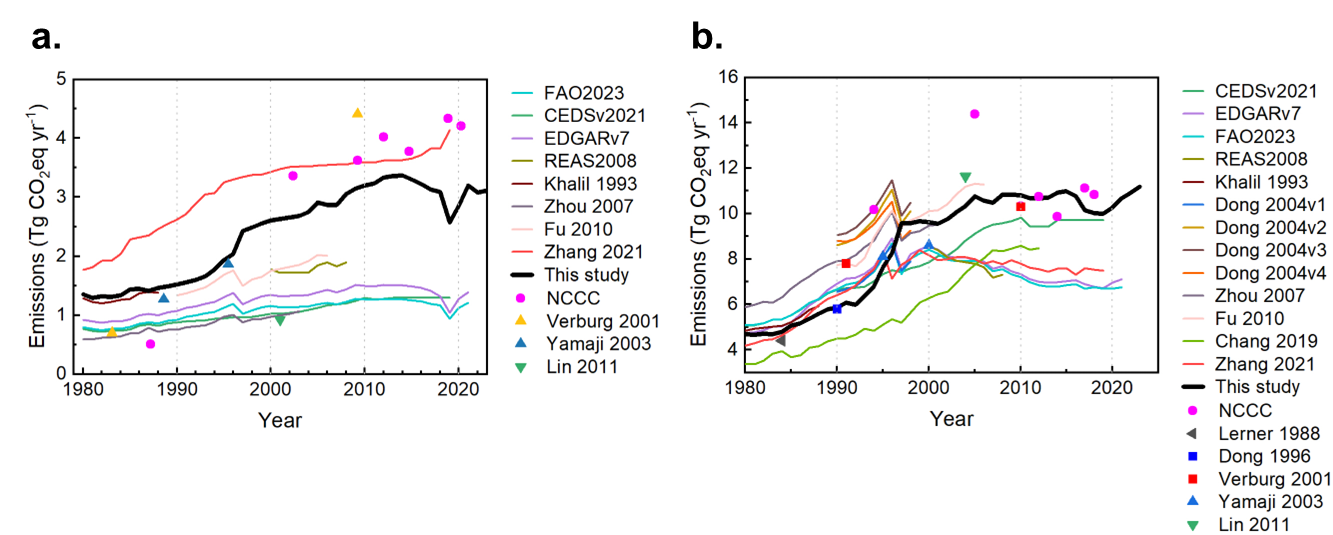


**Figure S2.** Comparisons of CH_4_ emissions from manure managements (a) and enteric fermentation (b) in this study with other datasets.


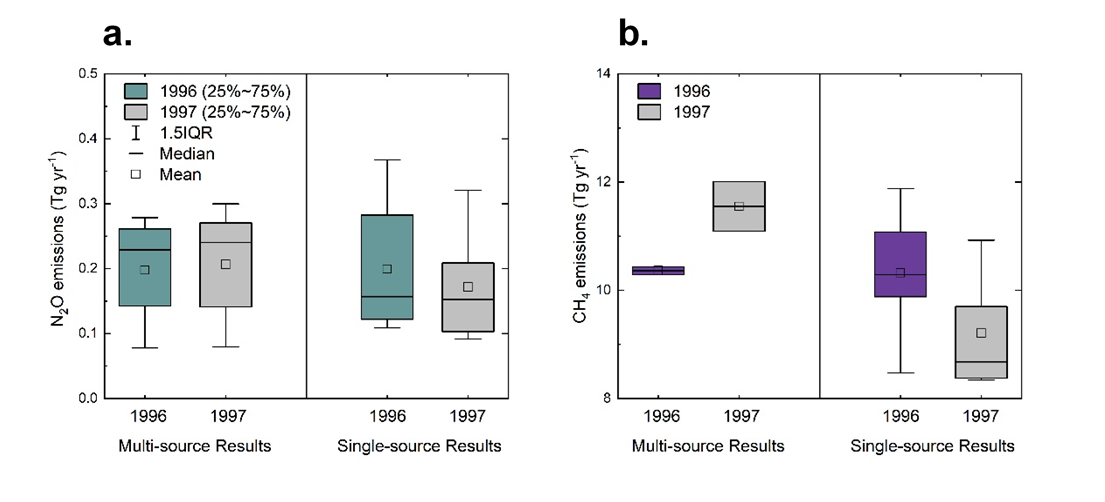


a

b

**Figure S3.** Comparisons of emission estimates in 1996 and 1997 from single data source and cross-referencing data source. (a) N_2_O estimates from 2 categories of studies; (b) CH_4_ estimates from the 2 types of studies. Details about the datasets can refer to Table S3-4.


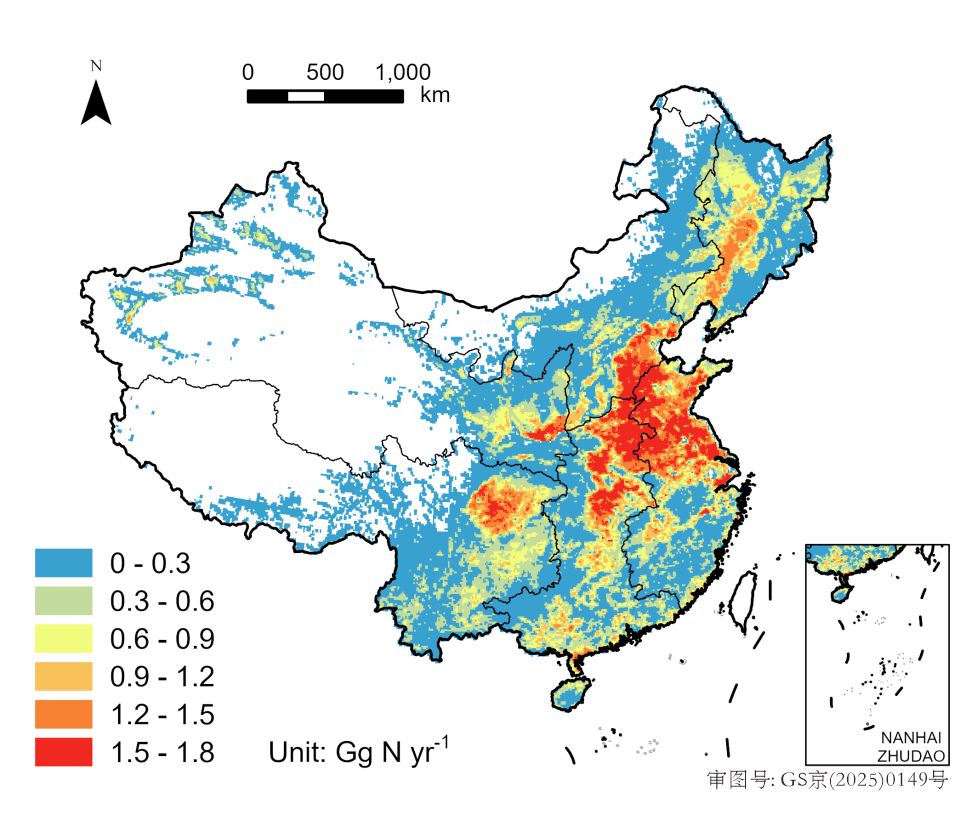


**Figure S4.** Spatial pattern of the nitrogen fertilizer inputs in China’s croplands. Data for Taiwan province is not included.


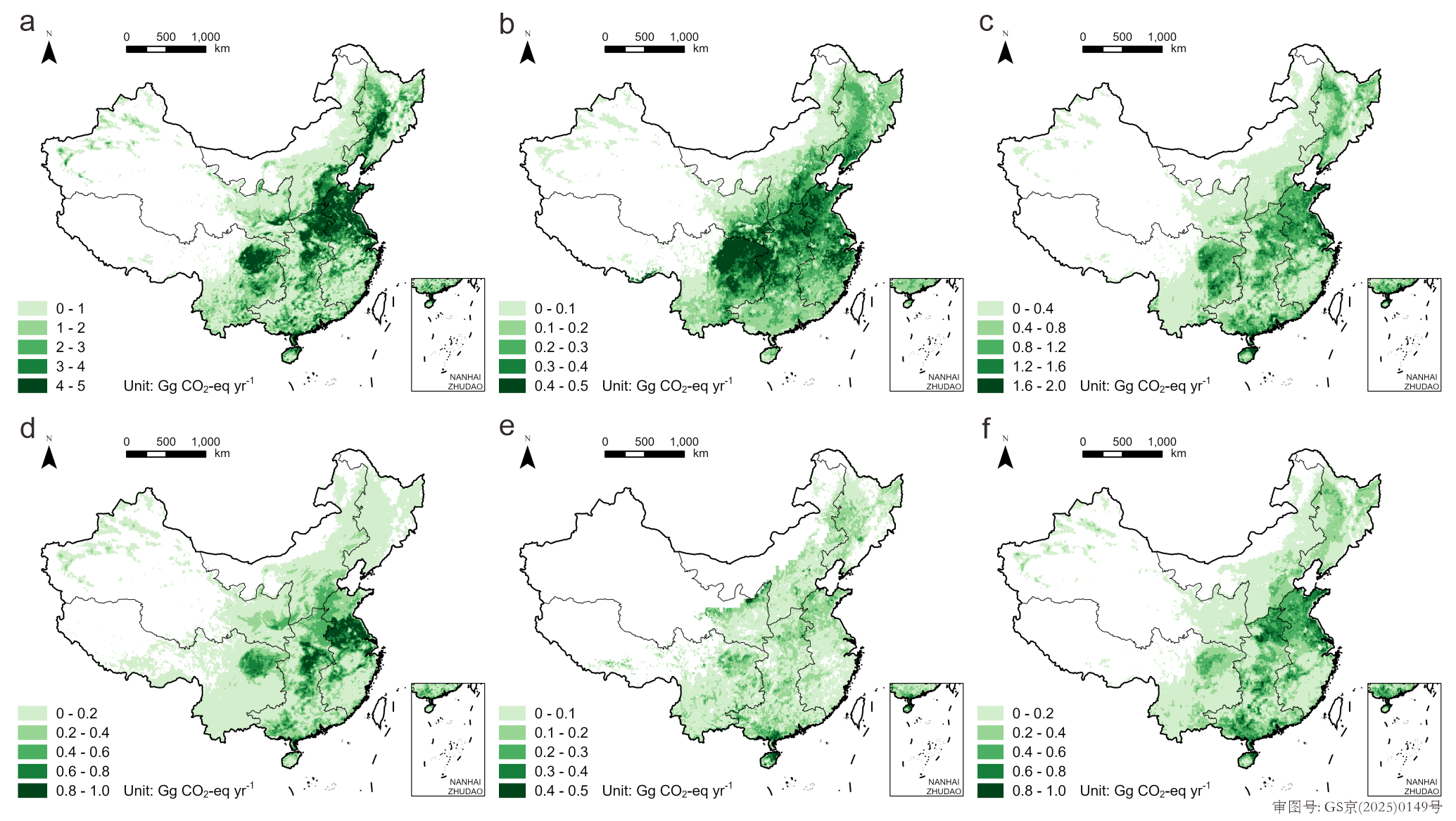


**Figure S5.** Spatial patterns of cropland N_2_O emissions induced by (a) N fertilization, (b) N deposition, (c) N mineralization, (d) N in crop residues, (e) N fertilization in pasture, and (f) N leaching. Notably, pasture nitrogen fertilization is typically categorized within the livestock sector. In this study, we employed the ML method to assess pasture N_2_O emission factors, aligning with the methodology used for cropland N_2_O emissions. Data for Taiwan province is not included.


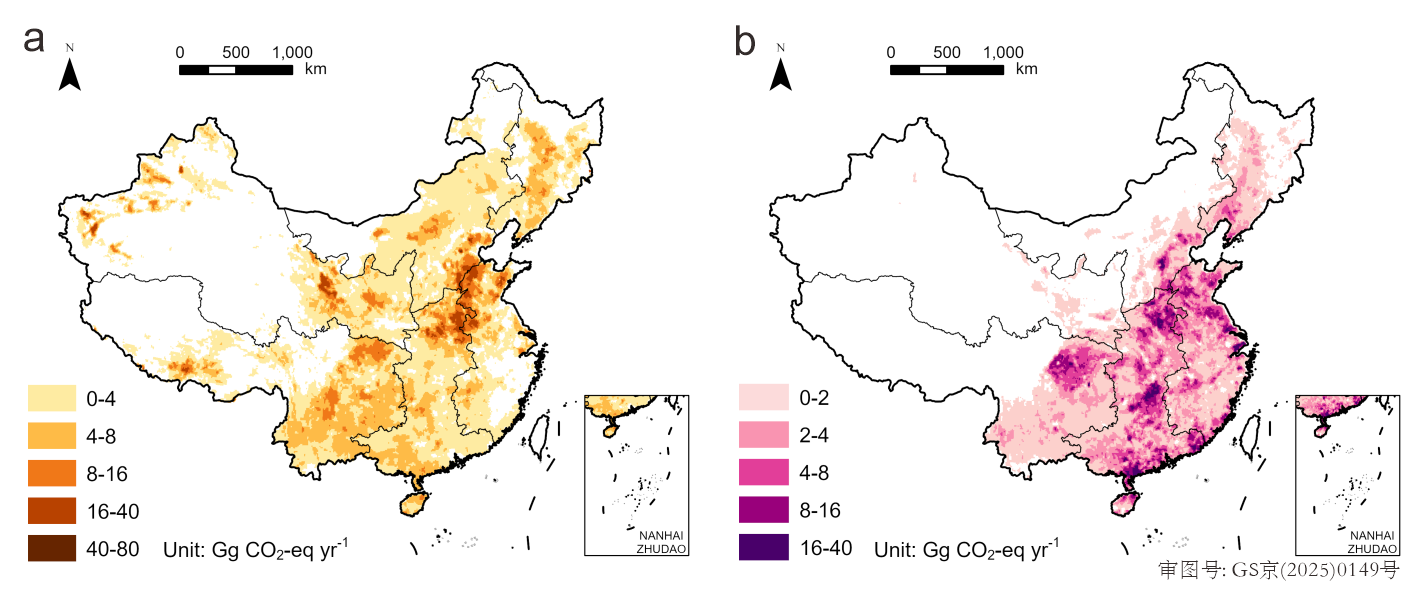


**Figure S6.** Non-CO_2_ GHG emissions from (a) ruminants and (b) monogastric animals. Data for Taiwan province is not included.


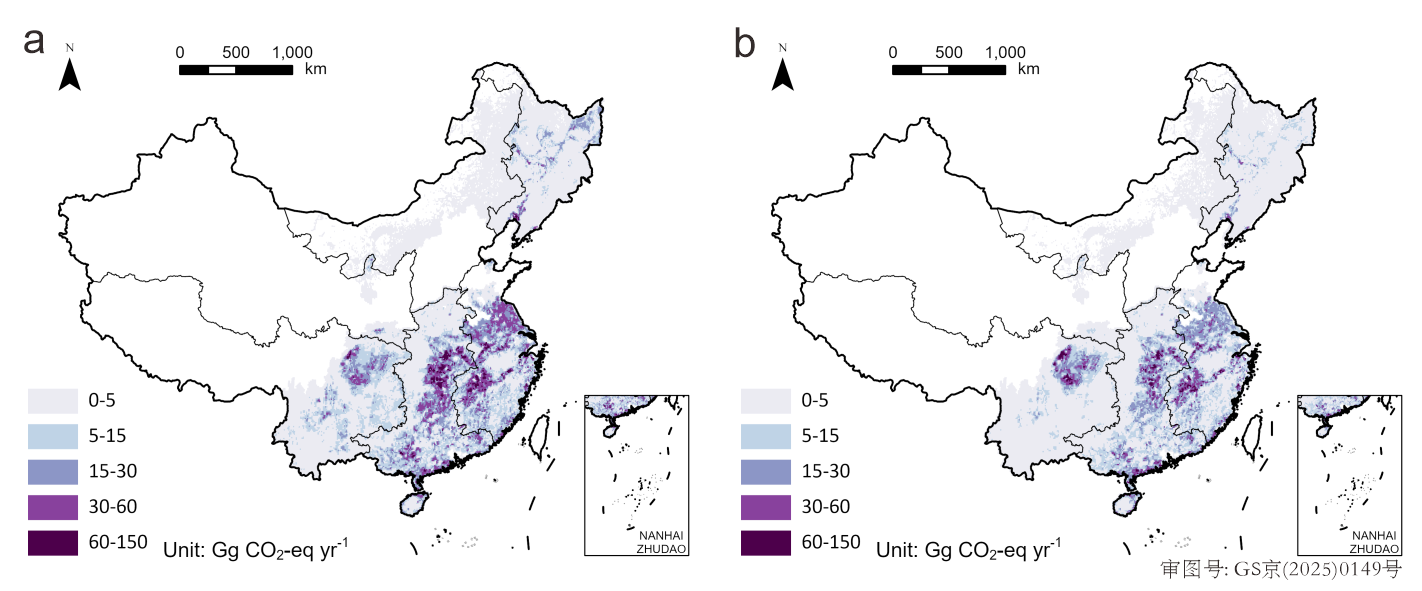


**Figure S7.** Spatial patterns of CH_4_ emissions of rice cultivation based on (a) IBIS-CH_4_ and (b) CH4MOD. Data for Taiwan province is not included.


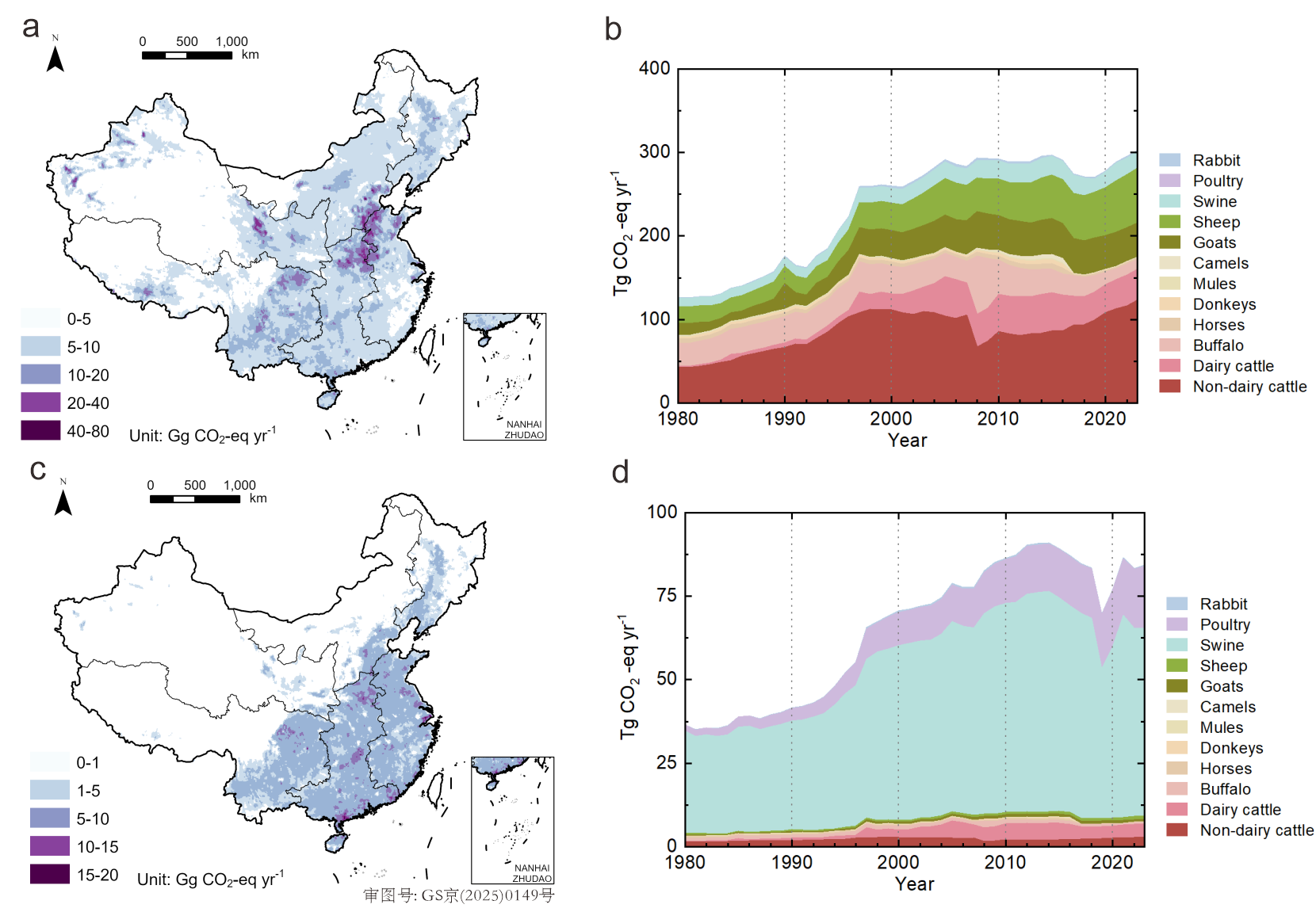


**Figure S8.** Spatiotemporal dynamics of CH_4_ emissions from enteric fermentation and manure management. (a) Spatial pattern of average results of CH_4_ emissions from enteric fermentation during 1980–2023. (b) Long-term CH_4_ emissions from enteric fermentation during 1980–2023 of 12 livestock categories. (c) Spatial pattern of average results of CH_4_ emissions from manure managements during 1980–2023. (d) Long-term CH_4_ emissions from manure managements during 1980–2023 of 12 livestock categories. Data for Taiwan province is not included.


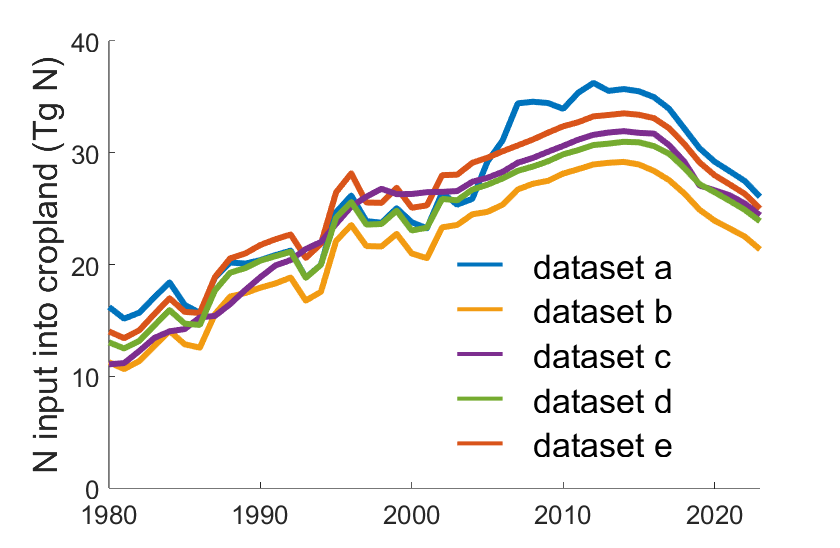


**Figure S9.** Temporal changes of N fertilizer applied to China’s croplands based on different datasets. Data sources for the five datasets are shown in Table S8.


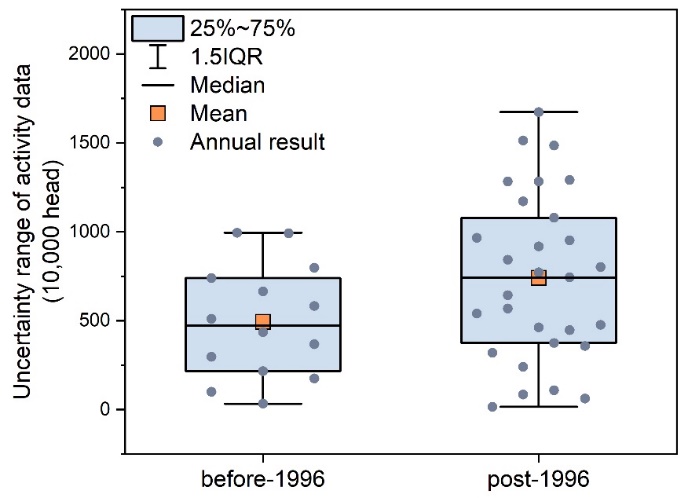


**Figure S10.** The variability in livestock activity data before and after 1996. This figure shows the uncertainty in livestock stock data arising from cross-referencing datasets in the cattle livestock category.


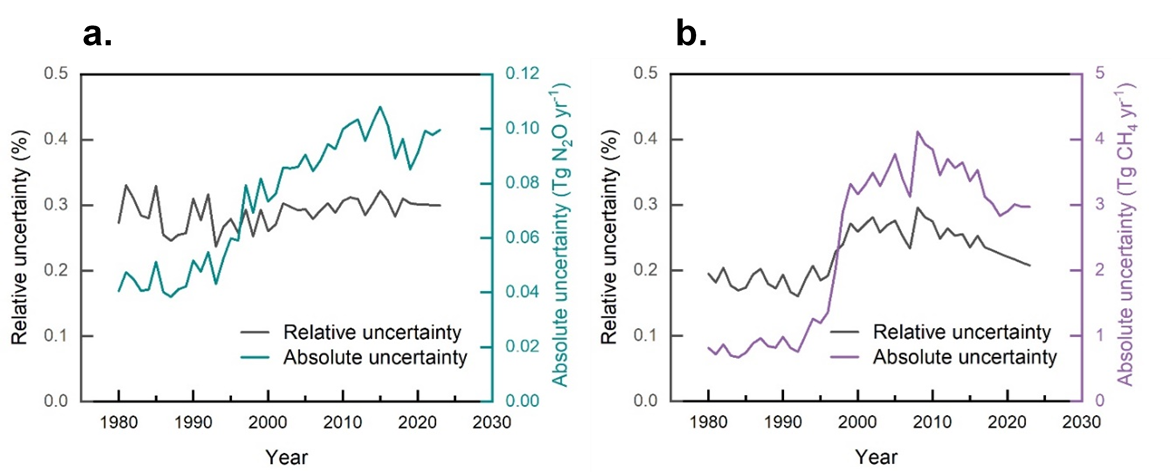


a

b

**Figure S11.** The relative uncertainty percentage (%) and absolute uncertainty (in Tg Gas yr^-1^) in (a) livestock N_2_O sectors, (b) livestock CH_4_ sectors.

**Section S3: Supplement tables**

**Table S1**. Comparison of data and methods for the cropland N_2_O emission estimation referred in Fig. 1a.

| **Sectors** | **Study** | **Method** | **EFs** | **Activity data level** | **Period** |
| --- | --- | --- | --- | --- | --- |
| Cropland  N_2_O | FAO [35] | Inventory (IPCC 2006 Tier1) | IPCC default | National | 1980–2020 |
|  | CEDS [36] | Inventory (IPCC 2006 Tier1) data-driven modification | IPCC default | National to Grid | 1980–2019 |
|  | EDGAR [37] | Inventory (IPCC 2006 Tier1) data-driven modification | IPCC default | National to Grid | 1980–2021 |
|  | PRIMAP HISTCR [38] PRIMAP HISTTP [38] | Inventory (IPCC 2006 Tier1) | IPCC default | National | 1980–2019 |
|  | Li et al., [39] | Inventory (IPCC 2006 Tier1) | Provincial | Provincial | 1996–2014 |
|  | Shang et al., [40] | Inventory (IPCC 2006 Tier1) | National | National | 1993–2011 |
|  | NGHGIs [34] | Inventory (IPCC 2006 Tier2) | Provincial | Provincial | 1994, 2005,  2010, 2012,  2014, 2017,  2018 |
|  | Cui et al., [41] | Data-driven model | Gridded | Grid | 1980–2017 |
|  | Wang et al., [42] | Data-driven model | Gridded | Grid | 1980–2014 |
|  | This study | Data-driven model | Gridded | Grid | 1980–2023 |

**Table S2**. Comparison of data and methods for the rice paddy CH_4_ emission estimation referred in Fig. 1b.

| **Sectors** | **Study** | **Method** | **EFs** | **Activity data level** | **Period** |
| --- | --- | --- | --- | --- | --- |
| Cropland  CH_4_ | FAO [35] | Inventory (IPCC 1996 Tier1) | IPCC default | National | 1980–2021 |
|  | CEDS [36] | Inventory (IPCC 2006 Tier1) data-driven modification | IPCC default | National to Grid | 1980–2019 |
|  | EDGAR [37] | Inventory (IPCC 2006 Tier1) | IPCC default | National to Grid | 1980–2021 |
|  | PRIMAP HISTCR [38] PRIMAP HISTTP [38] | Inventory (IPCC 2006 Tier1) | IPCC default | National | 1980–2019 |
|  | VISIT_Cao [43] VISIT_Wu [43] | Process-based model | - | Grid | 1980–2019 |
|  | PKU v1, v2 [44] | Inventory (IPCC 2006 Tier2) | Provincial | Provincial | 1980–2010,  2000–2019 |
|  | NGHGIs [34] | Process-based model | - | Grid | 1994, 2005,  2010, 2012,  2014, 2017,  2018 |
|  | This study | 2 Process-based models | - | Grid | 1980–2023 |

**Table S3**. Comparison of data and methods for the livestock N_2_O emission estimation referred in Fig. 1c.

| **Sectors** | **Study** | **Method** | **EFs** | **Activity data level** | **Period** |
| --- | --- | --- | --- | --- | --- |
| Livestock  N_2_O | FAO [45] | Inventory (IPCC 2006 Tier1) | IPCC default | National | 1980–2020 |
|  | CEDS [36] | Inventory (IPCC 2006 Tier1) data-driven modification | IPCC default | National to Grid | 1980–2019 |
|  | EDGAR [37] | Inventory (IPCC 2006 Tier1) data-driven modification | IPCC default | National to Grid | 1980–2021 |
|  | PRIMAP HISTCR [38] PRIMAP HISTTP | Inventory (IPCC 2006 Tier1) | IPCC default | National | 1980–2019 |
|  | REAS [46] | Inventory (IPCC 2006 Tier1) | National | National to Grid | 2000–2008 |
|  | EPA [47] | Inventory (IPCC 2006 Tier1) | IPCC default | National | 1990–2017 |
|  | Xu et al., [24] | Inventory (IPCC 2006 Tier2) N model | Provincial | County-level | 1980–2017 |
|  | Ding et al., [48] | Inventory (IPCC 2006 Tier2) | National | Provincial | 1990–2013 |
|  | Luo et al., [49] | Inventory (IPCC 2006 Tier2) N model | National | Provincial | 1980–2015 |
|  | Zhuang et al., [50] | Inventory (IPCC 2006 Tier2) | Provincial | Provincial | 2000–2015 |
|  | Li et al., [39] | Inventory (IPCC 2006 Tier1) | National | Provincial | 1996–2014 |
|  | Zhou et al., [51] | Inventory (IPCC 1996 Tier2) | National | National | 1980–2003 |
|  | NGHGIs [34] | Inventory (IPCC 2006 Tier2) | City-level | City-level | 1994, 2005,  2010, 2012,  2014, 2017,  2018 |
|  | Wu et al., [52] | Inventory (IPCC 2006 Tier2) | National | National | 1985, 1990,  1995, 2000,  2005, 2010,  2015 |
|  | This study | Inventory (IPCC 2019 Tier2) | Provincial | County-level to grid | 1980–2023 |

**Table S4**. Comparison of data and methods for the livestock CH_4_ emission estimation referred in Fig. 1d. Methane emissions from enteric fermentation and manure managements were compared separately.

| **Sectors** | **Study** | **Method** | **EFs** | **Activity data level** | **Period** |
| --- | --- | --- | --- | --- | --- |
| Enteric fermentation | FAOSTAT [45] | Inventory (IPCC 2006 Tier1) | IPCC default | National | 1980–2020 |
|  | CEDS [36] | Inventory (IPCC 2006 Tier1) | IPCC default | National to Grid | 1980–2019 |
|  | EDGAR [37] | Inventory (IPCC 2006 Tier2) | National | National to Grid | 1980–2021 |
|  | REAS [46] | Inventory (IPCC 2006 Tier1) | IPCC default | National to Grid | 2000–2008 |
|  | Khalil et al., [53] | Inventory (IPCC 1996 Tier1) | National | National | 1980–1988 |
|  | Lerner et al., [54] | Inventory (IPCC 1996 Tier1) | IPCC default | Provincial | 1984 |
|  | 2019T1 Chang et al., [55] | Inventory (IPCC 2019 Tier1) | IPCC default | National to Grid | 2000-2018 |
|  | Dong et al., [56] | Inventory (IPCC 1996 Tier2) | National | Provincial | 1990–1998 |
|  | Zhou et al., [51] | Inventory (IPCC 1996 Tier2) | National | National | 1980–2003 |
|  | Fu et al., [57] | Inventory (IPCC 2006 Tier2) | National | Provincial | 1990–2006 |
|  | Chang et al., [58] | Inventory (IPCC 2006 Tier2) | National | National | 1980–2012 |
|  | Zhang et al., [20] | Inventory (IPCC 2019 Tier2) | National | Provincial to grid | 1980–2019 |
|  | NGHGIs [34] | Inventory (IPCC 2006 Tier2) | City-level | City-level | 1994, 2005,  2010, 2012,  2014, 2017,  2018 |
|  |  |  |  |  |  |
|  | Verburg et al., [59] | Inventory (IPCC 1996 Tier2) | National | Grid | 1991, 2010 |
|  | Yamaji et al., [60] | Inventory (IPCC 1996 Tier2) | National | Provincial to grid | 1995, 2000 |
|  | Lin et al., [61] | Inventory (IPCC 2006 Tier2) | National | County | 2004 |
|  | 2019MT  Chang et al., [55] | Inventory (IPCC 2019 Tier2) | National | National to Grid | 2000-2018 |
|  | This study | Inventory (IPCC 2019 Tier2) | Provincial | County-level to grid | 1980–2023 |
| Manure management | FAOSTAT [45] | Inventory (IPCC 2006 Tier1) | IPCC default | National | 1980–2020 |
|  | CEDS [36] | Inventory (IPCC 2006 Tier1) data-driven modification | IPCC default | National to Grid | 1980–2019 |
|  | EDGAR [37] | Inventory (IPCC 2006 Tier1) data-driven modification | National | National to Grid | 1980–2021 |
|  | PRIMAP HISTCR [38] PRIMAP HISTTP [38] | Inventory (IPCC 2006 Tier1) | IPCC default | National | 1980–2019 |
|  | REAS [46] | Inventory (IPCC 2006 Tier1) | IPCC default | National to Grid | 2000–2008 |
|  | Khalil et al., [53] | Inventory (IPCC 1996 Tier1) | National | National | 1980–1988 |
|  | 2019T1 Chang et al., [55] | Inventory (IPCC 2019 Tier1) | IPCC default | National to Grid | 2000-2018 |
|  | Zhou et al., [51] | Inventory (IPCC 1996 Tier2) | National | National | 1980–2003 |
|  | Fu et al., [57] | Inventory (IPCC 2006 Tier2) | National | Provincial | 1990–2006 |
|  | Zhang et al., [20] | Inventory (IPCC 2019 Tier2) | National | Provincial to grid | 1980–2019 |
|  | NGHGIs [34] | Inventory (IPCC 2006 Tier2) | City-level | City-level | 1994, 2005,  2010, 2012,  2014, 2017,  2018 |
|  | Verburg et al., [59] | Inventory (IPCC 1996 Tier2) | National | Grid | 1991, 2010 |
|  | Yamaji et al., [60] | Inventory (IPCC 1996 Tier2) | National | Provincial to grid | 1995, 2000 |
|  | Lin et al., [61] | Inventory (IPCC 2006 Tier2) | National | County | 2004 |
|  | 2019MT  Chang et al., [55] | Inventory (IPCC 2019 Tier2) | National | National to Grid | 2000-2018 |
|  | This study | Inventory (IPCC 2019 Tier2) | Provincial | County-level to grid | 1980–2023 |

**Table S5.** Average results of application rate (*%*) from different manure management systems in main animal types.

| **Manure management** | **non-dairy cattle** | **dairy cattle** | **buffalo** | **swine** | **sheep** | **goats** |
| --- | --- | --- | --- | --- | --- | --- |
| *Solid Storage* | 15.3 | 18 | 8.2 | 19.4 | 18.1 | 30.6 |
| *Liquid/Slurry* | 24.3 | 21.9 | 30.8 | 34.1 | 15.9 | 24.6 |
| *Anaerobic digester* | 2.2 | 5.1 | 1.7 | 14 | 0.1 | 0.5 |
| *Aerobic treatment* | 0 | 0 | 0 | 0.5 | 0.1 | 0 |
| *Compost* | 25 | 17 | 41 | 25 | 15.3 | 18 |
| *Pasture/Range* | 26.9 | 30.2 | 16.7 | 0 | 49.5 | 24.5 |
| *Burned for Fuel* | 0.1 | 0 | 0.4 | 0 | 0 | 0 |
| *Other* | 6.2 | 7.8 | 1.2 | 7 | 1 | 1.8 |

**Table S6**. Nitrogen loss coefficients (*%*) in different manure management stages from different productions systems.

| **Manure**  **management stage** | **Production system** | **N loss type** | | | | |
| --- | --- | --- | --- | --- | --- | --- |
|  |  | *NH_3_* | *N_2_O from slurry* | *N_2_O from solids* | *N_2_* | *NO_3_^-^ leaching loss rate* |
| Housing | Traditional | 30 | 2.17 | 0.5 | 5 | 0 |
|  | Medium | 16 | 2.17 | 0.5 | 5 | 0 |
|  | Industrial | 10 | 2.17 | 0.5 | 5 | 0 |
| Storage | Traditional | 30 | 2.17 | 0.5 | 5 | 5~20 |
|  | Medium | 16 | 2.17 | 0.5 | 5 | 0~10 |
|  | Industrial | 10 | 2.17 | 0.5 | 5 | 0 |
| Treatment | Recycling | 18 | 1.8 | 1.8 | 4.2 | 12 |
|  | Composting | 28.37 | 0 | 0.26 | 5 | 0 |
|  | Nonseparation | 28 | 0.3 | 0.3 | 5 | 0 |
|  | Separation | 18.67 | 0.3 | 0.3 | 5 | 0 |
| Grazing | - | 16 | 0.5 | 0.5 | 5 | 20 |

**Table S7**. Parameters for estimating manure nitrogen applied to croplands across livestock systems, types, and climate zones. It should be noted that we only considered manure fertilizer applied to cropland in mixing farming regions referred from Herrero et al. (2013).

| **Production system** | **Animal type** | **Climate zone** | **Frac (*%*)** |
| --- | --- | --- | --- |
| Traditional and medium production system | dairy cattle | Temperate | 19.7 |
|  |  | Humid | 13.4 |
|  |  | Arid | 9.6 |
|  | non-dairy cattle, buffalo | Temperate | 15.9 |
|  |  | Humid | 9.3 |
|  |  | Arid | 10.7 |
|  | Small Ruminant | Temperate | 3.8 |
|  |  | Humid | 3.3 |
|  |  | Arid | 14.3 |
|  | Swine | - | 27.0 |
|  | Poultry | - | 12.0 |
| Industrial production systems | dairy cattle | Temperate | 31.7 |
|  |  | Humid | 24.9 |
|  |  | Arid | 15.4 |
|  | non-dairy cattle, buffalo | Temperate | 15.9 |
|  |  | Humid | 13.8 |
|  |  | Arid | 9.8 |
|  | Small Ruminant | Temperate | 28.9 |
|  |  | Humid | 22.3 |
|  |  | Arid | 10.7 |
|  | Swine | - | 29.0 |
|  | Poultry | - | 36.0 |

**Table S8**. Five datasets of N fertilization into the cropland as in Fig. S9.

| Dataset | Source | | |
| --- | --- | --- | --- |
|  | *Nrate_chem_* | *Nrate_man_* | *area* |
| dataset a | Lu et al., 2017 [62] | Zhang et al., 2017 [63] | Yu et al., 2021 [64] |
| dataset b | Tian et al., 2022 [1] | | |
| dataset c | Yu et al., 2022 [65] | Zhang et al., 2017 [63] | Yu et al., 2021 [64] |
| dataset d | Adalibieke et al., 2023 [66] | | |
| dataset e | Li et al., in prep. [3]  based on NBSC [12] | Gao et al., in prep. [67] | Li et al., in prep. [3]  based on NBSC [12] |

**References:**

1. Tian H, Bian Z, Shi H *et al*. History of anthropogenic Nitrogen inputs (HaNi) to the terrestrial biosphere: a 5 arcmin resolution annual dataset from 1860 to 2019. *Earth Syst Sci Data* 2022; **14**: 4551-68.

2. Liang M, Zhou Z, Ren P *et al*. Four decades of full-scale nitrous oxide emission inventory in China. *National Science Review* 2024: nwad285.

3. Li Z, et al. (in preparation). Fertilization-induced N2O emissions in China's croplands during 1980-2022.

4. Zhou M and Butterbach-Bahl K. Assessment of nitrate leaching loss on a yield-scaled basis from maize and wheat cropping systems. *Plant and Soil* 2014; **374**: 977-91.

5. Aliyu G, Luo J, Di HJ *et al*. Nitrous oxide emissions from China's croplands based on regional and crop-specific emission factors deviate from IPCC 2006 estimates. *Science of The Total Environment* 2019; **669**: 547-58.

6. Cui X, Zhou F, Ciais P *et al*. Global mapping of crop-specific emission factors highlights hotspots of nitrous oxide mitigation. *Nature Food* 2021.

7. Huang Y, Zhang W, Zheng X *et al*. Estimates of methane emissions from Chinese rice paddies by linking a model to GIS database. *Acta Ecologica Sinica* 2006; **26**: 980-87.

8. Huang Y, Sass RL and Fisher FMJ. A semi‐empirical model of methane emission from flooded rice paddy soils. *Global Change Biology* 1998; **4**: 247-68.

9. Huang Y, Zhang W, Zheng X *et al*. Modeling methane emission from rice paddies with various agricultural practices. *Journal of Geophysical Research: Atmospheres* 2004; **109**.

10. Xie B, Zhou Z, Zheng X *et al*. Modeling methane emissions from paddy rice fields under elevated atmospheric carbon dioxide conditions. *Advances in Atmospheric Sciences* 2010; **27**: 100-14.

11. Muñoz-Sabater J, Dutra E, Agustí-Panareda A *et al*. ERA5-Land: a state-of-the-art global reanalysis dataset for land applications. *Earth Syst Sci Data* 2021; **13**: 4349-83.

12. National Statistical Database (1999-2022). National Bureau of Statistics of China. Available at <http://data.stats.gov.cn/easyquery.htm?cn=C01>

13. Zhang W, Yu Y, Huang Y *et al*. Modeling methane emissions from irrigated rice cultivation in China from 1960 to 2050. *Global Change Biology* 2011; **17**: 3511-23.

14. Zhang W, Yu Y, Li T *et al*. Net Greenhouse Gas Balance in China’s Croplands over the Last Three Decades and Its Mitigation Potential. *Environmental Science & Technology* 2014; **48**: 2589-97.

15. Song C, Luan J, Xu X *et al*. A Microbial Functional Group-Based CH4 Model Integrated Into a Terrestrial Ecosystem Model: Model Structure, Site-Level Evaluation, and Sensitivity Analysis. *Journal of Advances in Modeling Earth Systems* 2020; **12**: e2019MS001867.

16. Lu H, Yuan W and Chen X. A Processes-Based Dynamic Root Growth Model Integrated Into the Ecosystem Model. *Journal of Advances in Modeling Earth Systems* 2019; **11**: 4614-28.

17. Barison J and Uphoff N. Rice yield and its relation to root growth and nutrient-use efficiency under SRI and conventional cultivation: an evaluation in Madagascar. *Paddy and Water Environment* 2011; **9**: 65-78.

18. IPCC. 2019 Refinement to the 2006 IPCC Guidelines for National Greenhouse Gas Inventories. Hyama: Institute for Global Environmental Strategies, 2019.

19. China Agriculture Yearbook (1980-2021). China Agricultural Yearbook Editorial Committee. Available at <https://cnki.ctbu.edu.cn/CSYDMirror/trade/yearbook/Single/N2022030154?z=Z009>

20. Zhang L, Tian H, Shi H *et al*. Methane emissions from livestock in East Asia during 1961-2019. *Ecosystem Health and Sustainability* 2021; **7**.

21. National Agricultural Census of China (1996, 2006, 2016). National Bureau of Statistics of China. Available at <https://www.stats.gov.cn/sj/tjgb/nypcgb/qgnypcgb/>

22. Notice on releasing the revised results of the second national agricultural census of Beijing on agricultural historical data. Beijing Municipal Bureau of Statistics. 2008. Available at <https://tjj.beijing.gov.cn/tjsj_31433/tjgb_31445/npgb_31448/202002/t20200216_1643320.html>

23. Zhang C, Liu S, Wu S *et al*. Rebuilding the linkage between livestock and cropland to mitigate agricultural pollution in China. *Resources, Conservation and Recycling* 2019; **144**: 65-73.

24. Xu P, Houlton BZ, Zheng Y *et al*. Policy-enabled stabilization of nitrous oxide emissions from livestock production in China over 1978–2017. *Nature Food* 2022; **3**: 356-66.

25. Bai Z, Fan X, Jin X *et al*. Relocate 10 billion livestock to reduce harmful nitrogen pollution exposure for 90% of China’s population. *Nature Food* 2022; **3**: 152-60.

26. Wang M, Ma L, Strokal M *et al*. Hotspots for Nitrogen and Phosphorus Losses from Food Production in China: A County-Scale Analysis. *Environmental Science & Technology* 2018; **52**: 5782-91.

27. Zhu Z, Zhang X, Dong H *et al*. Integrated livestock sector nitrogen pollution abatement measures could generate net benefits for human and ecosystem health in China. *Nature Food* 2022; **3**: 161-68.

28. Duan J, Ren C, Wang S *et al*. Consolidation of agricultural land can contribute to agricultural sustainability in China. *Nature Food* 2021; **2**: 1014-22.

29. Gilbert M, Cinardi G, Da Re D *et al*. Global distribution data for cattle, buffaloes, horses, sheep, goats, pigs, chickens and ducks in 2015 (5 minutes of arc). Harvard Dataverse. 2022. Available at <https://doi.org/10.7910/DVN/SXHLF3>

30. Gilbert M, Nicolas G, Cinardi G *et al*. Global distribution data for cattle, buffaloes, horses, sheep, goats, pigs, chickens and ducks in 2010. 2018. Available at <https://doi.org/10.1038/sdata.2018.227>

31. Yu J, Peng S, Chang J *et al*. Inventory of methane emissions from livestock in China from 1980 to 2013. *Atmospheric Environment* 2018; **184**: 69-76.

32. Xu P, Liao Y, Zheng Y *et al*. Northward shift of historical methane emission hotspots from the livestock sector in China and assessment of potential mitigation options. *Agricultural and Forest Meteorology* 2019; **272-273**: 1-11.

33. Opio C, Gerber P, Mottet A *et al*. Greenhouse gas emissions from ruminant supply chains-A global life cycle assessment. Food and Agriculture Organization of the United Nations (FAO), 2013.

34. NGHGI. National communication (NC1-3) and Biennial update report (BUR1-3). Ministry of Ecology and Environment of the People's Republic of China. 2004, 2012, 2016, 2018, 2023. Available at <https://www.mee.gov.cn/ywdt/hjywnews/202312/t20231229_1060290.shtml>

35. FAO. FAOSTAT Climate Change: Agrifood systems emissions, Emissions from crops. Rome, Italy. 2023. Available at <https://www.fao.org/faostat/en/#data/GCE>

36. McDuffie EE, Smith SJ, O'Rourke P *et al*. A global anthropogenic emission inventory of atmospheric pollutants from sector- and fuel-specific sources (1970–2017): an application of the Community Emissions Data System (CEDS). *Earth System Science Data* 2020; **12**: 3413-42.

37. Crippa M, Guizzardi D, Solazzo E *et al*. GHG emissions of all world countries-2021 Report. Luxembourg: Publications Office of the European Union, 2021.

38. Gütschow J, Günther A and Pflüger M. The PRIMAP-hist national historical emissions time series (1750-2019) v2.3.1 (2.3.1). Zenodo. 2021. Available at <https://doi.org/10.5281/zenodo.4479171>

39. Li N, Shang L, Yu Z *et al*. Estimation of agricultural greenhouse gases emission in interprovincial regions of China during 1996–2014. *Natural Hazards* 2020; **100**: 1037-58.

40. Shang J, Yang G and Fawen. Y. Agricultural greenhouse gases emissions and influencing factors in China. *Chinese Journal of Eco-Agriculture* 2015; **23**: 354-64.

41. Cui X, Shang Z, Xia L *et al*. Deceleration of Cropland-N2O Emissions in China and Future Mitigation Potentials. *Environmental Science & Technology* 2022; **56**: 4665-75.

42. Wang Q, Zhou F, Shang Z *et al*. Data-driven estimates of global nitrous oxide emissions from croplands. *National Science Review* 2020; **7**: 441-52.

43. Ito A and Inatomi M. Use of a process-based model for assessing the methane budgets of global terrestrial ecosystems and evaluation of uncertainty. *Biogeosciences* 2012; **9**: 759-73.

44. Peng S, Piao S, Bousquet P *et al*. Inventory of anthropogenic methane emissions in mainland China from 1980 to 2010. *Atmospheric Chemistry and Physics* 2016; **16**: 14545-62.

45. FAO. FAOSTAT Climate Change: Agrifood systems emissions, Emissions from Livestock. 2023. Available at <https://www.fao.org/faostat/en/#data/GLE>

46. Kurokawa J and Ohara T. Long-term historical trends in air pollutant emissions in Asia: Regional Emission inventory in ASia (REAS) version 3. *Atmospheric Chemistry and Physics* 2020; **20**: 12761-93.

47. USEPA. Global Non-CO2 Greenhouse Gas Emission Projections & Marginal Abatement Cost Analysis: Methodology Documentation. U.S. Environmental Protection Agency Office of Atmospheric Programs, Climate Change Division Washington, DC. 2019. Available at <https://www.epa.gov/sites/default/files/2019-09/documents/nonco2_methodology_report.pdf>

48. Ding T, Ning Y and Zhang Y. Estimation of greenhouse gas emissions in China 1990–2013. *Greenhouse Gases: Science and Technology* 2017; **7**: 1097-115.

49. Luo Z, Lam SK, Fu H *et al*. Temporal and spatial evolution of nitrous oxide emissions in China: Assessment, strategy and recommendation. *Journal of Cleaner Production* 2019; **223**: 360-67.

50. Zhuang M, Lu X, Caro D *et al*. Emissions of non-CO2 greenhouse gases from livestock in China during 2000–2015: Magnitude, trends and spatiotemporal patterns. *Journal of Environmental Management* 2019; **242**: 40-45.

51. Zhou J, Jiang M and Chen G. Estimation of methane and nitrous oxide emission from livestock and poultry in China during 1949–2003. *Energy Policy* 2007; **35**: 3759-67.

52. Wu H, Wang S, Gao L *et al*. Nutrient-derived environmental impacts in Chinese agriculture during 1978–2015. *Journal of Environmental Management* 2018; **217**: 762-74.

53. Khalil MAK, Shearer MJ and Rasmussen RA. Methane sources in China: Historical and current emissions. *Chemosphere* 1993; **26**: 127-42.

54. Lerner J, Matthews E and Fung I. Methane emission from animals: A Global High-Resolution Data Base. *Global Biogeochemical Cycles* 1988; **2**: 139-56.

55. Chang J, Peng S, Yin Y *et al*. The Key Role of Production Efficiency Changes in Livestock Methane Emission Mitigation. *AGU Advances* 2021; **2**.

56. Dong H, Tao X, Xin H *et al*. Comparison of enteric methane emissions in China for different IPCC estimation methods and production schemes. *Transactions of the ASAE* 2004; **47**: 2051-57.

57. Fu C and Yu G. Estimation and Spatiotemporal Analysis of Methane Emissions from Agriculture in China. *Environmental Management* 2010; **46**: 618-32.

58. Chang J, Peng S, Ciais P *et al*. Revisiting enteric methane emissions from domestic ruminants and their δ13CCH4 source signature. *Nature Communications* 2019; **10**: 3420.

59. Verburg PH and Van Der Gon HACD. Spatial and temporal dynamics of methane emissions from agricultural sources in China. *Global Change Biology* 2001; **7**: 31-47.

60. Yamaji K, Ohara T and Akimoto H. A country-specific, high-resolution emission inventory for methane from livestock in Asia in 2000. *Atmospheric Environment* 2003; **37**: 4393-406.

61. Lin Y, Zhang W and Huang Y. Estimating Spatiotemporal Dynamics of Methane Emissions from Livestock in China. *Environmental Science* 2011; **32**: 2212-20.

62. Lu C and Tian H. Global nitrogen and phosphorus fertilizer use for agriculture production in the past half century: shifted hot spots and nutrient imbalance. *Earth Syst Sci Data* 2017; **9**: 181-92.

63. Zhang B, Tian H, Lu C *et al*. Global manure nitrogen production and application in cropland during 1860–2014: a 5 arcmin gridded global dataset for Earth system modeling. *Earth Syst Sci Data* 2017; **9**: 667-78.

64. Yu Z, Jin X, Miao L *et al*. A historical reconstruction of cropland in China from 1900 to 2016. *Earth Syst Sci Data* 2021; **13**: 3203-18.

65. Yu Z, Liu J and Kattel G. Historical nitrogen fertilizer use in China from 1952 to 2018. *Earth Syst Sci Data* 2022; **14**: 5179-94.

66. Adalibieke W, Cui X, Cai H *et al*. Global crop-specific nitrogen fertilization dataset in 1961–2020. *Scientific Data* 2023; **10**: 617.

67. Gao Y, et al. (in preparation). Near-real-time gridded livestock GHG emissions in China since 1980.
